# Structural defects in male meiotic cell division lead to reproductive genome instability and disturbed microsporogenesis in banana (*Musa* spp.)

**DOI:** 10.3389/fpls.2026.1923014

**Published:** 2026-09-30

**Authors:** Astrid Severyns, Aybars Koç, Steven B. Janssens, Rony Swennen, Nico De Storme

**Affiliations:** 1 Laboratory of Plant Genetics and Crop Improvement, Division of Crop Biotechnics, Department of Biosystems, KU Leuven, Heverlee, Belgium; 2 Meise Botanic Garden, Meise, Belgium; 3 Leuven Plant Institute, KU Leuven, Heverlee, Belgium; 4 Department of Biology, KU Leuven, Heverlee, Belgium; 5 Laboratory of Tropical Crop Improvement, Division of Crop Biotechnics, Department of Biosystems, KU Leuven, Heverlee, Belgium; 6 International Institute of Tropical Agriculture, Banana Breeding, Namulonge-Sendusu, Kampala, Uganda

**Keywords:** 2n gamete, banana, meiotic restitution, meiotic stability, *Musa*, polyploidy, structural heterozygosity

## Abstract

**Background:**

Reduced fertility in banana poses issues towards the genetic improvement of the crop. This study investigates male meiotic instability in banana, taking into account how it is shaped by genomic determinants, like ploidy, structural heterozygosity, and multiple ancestor contributions, and how it relates to decreased male fertility. In addition, the prevalence of meiotic restitution and related 2n gamete formation will be assessed due to its pivotal role in the emergence of current triploid landraces and its relevance for current triploid breeding.

**Results:**

Wild diploid *Musa* species are generally expected to undergo regular meiosis, yet several displayed significant polyad formation, particularly *M. acuminata* ‘Banksii’ and *M. balbisiana* ‘Eti Kehel’, due to univalents and lagging chromosomes in meiosis I. Polyad formation was more pronounced in cultivated diploids; for instance, ‘Pisang Jari Buaya’ exhibited extensive lagging chromosomes in both anaphase I and II, suggesting spindle attachment defects. A baseline of ~10% polyads was observed in triploid and tetraploid genotypes, while the triploid ‘Williams Bell’ showed elevated polyad formation linked to high levels of asynapsis and persistent univalents. Only a few genotypes across ploidy levels showed evidence of meiotic restitution, indicating that 2n pollen formation may be driven more by mutations in meiotic genes than by structural genome variation. Notably, the diploid ‘Pisang Lilin’ consistently produced 2n pollen, potentially through both premeiotic doubling and restitution. Although the influence of hybrid genome composition on meiotic behavior was inconclusive, increased hybridity correlated with reduced pollen production. Meiotic stability in banana could be related to mature pollen size. Pollen larger than 120 µm was associated with meiotic restitution in cultivated diploids, while pollen smaller than 60 µm correlated with polyad formation in wild diploids.

**Conclusion:**

This study contributes to our understanding of how genomic determinants affect meiotic stability and male fertility in banana and can aid in high-throughput assessment of meiotic stability in banana on the mature pollen level.

## Background

In the currently accepted model of banana domestication, humans facilitated contact between fertile but geographically isolated *M. acuminata* (AA) subspecies (De Langhe et al., 2009; Perrier et al., 2011). This interaction led to hybridizations between ssp. *banksii, burmannica, errans, malaccensis, microcarpa, siamea, truncata*, *zebrina* and *halabanensis*, initially producing diploid AA hybrids with reduced fertility due to genetic incompatibilities accumulated through their separate evolution. However, these hybrids also showed occasional formation of diploid (2n) gametes due to chromosomal abnormalities and meiotic disruptions (Subbaraya et al., 2011), thereby establishing a natural way to restore male fertility. Subsequent hybridizations, either within *M. acuminata* or involving other *Musa* species, such as *Musa balbisiana* (BB), *Musa schizocarpa* (SS) and *Musa textilis* (TT), gave rise to diploid and triploid banana cultivars, which were further diversified through additional cross-hybridizations, but also through long-term and large-scale vegetative propagation leading to the accumulation of somatic mutations, under selection for traits such as seedlessness and parthenocarpy (Martin et al., 2023a). Depending on the original *Musa* species contributing to their genome and their respective ploidy level, cultivars were grouped into following categories; AAcv, AAA, AB, AAB, ABB, AS, AT, and AAT (Simmonds and Shepherd, 1955; D’Hont et al., 2000).

Banana breeding is complicated by reduced male and female fertility and low genetic variability due to low levels of recombination (Ortiz and Swennen, 2014; Martin et al., 2020). Associated defects in meiotic cell division such as poor homoeologous chromosome pairing and uneven chromosome segregation, result in the formation of infertile aneuploid spores. In triploids the uneven chromosome number further adds to the unbalanced chromosome segregation (Dodds and Simmonds, 1946; Shepherd, 1999). Additionally, reciprocal translocations in diploid heterozygous parents reduce meiotic recombination surrounding the translocated regions, thereby leading to skewed segregation and linkage among these regions, or even the formation of meiosis I (MI) univalents (Martin et al., 2020). Six major reciprocal translocations have been identified within *M. acuminata* subspecies (Martin et al., 2017; Dupouy et al., 2019; Martin et al., 2020), along with a translocation and an inversion event between *M. acuminata* and *M. balbisiana* (Baurens et al., 2018; Šimoníková et al., 2019), many of which were passed on to cultivars (Němečková et al., 2018; Martin et al., 2020; Martin et al., 2023b; Beránková et al., 2024).

Recent studies revealed that *Musa* cultivars exhibit complex inter(sub)specific chromosome mosaics, with an overall contribution of a total of eleven genetic pools. Individual diploid and triploid cultivars have genome mosaics involving three up to possibly seven contributors (Baurens et al., 2018; Martin et al., 2020; Martin et al., 2023b). A majority of banana cultivars (70.5%) was found to be structurally heterozygous, due to one to four *M. acuminata* translocations. Translocations 1/4 and 3/8, linked to *malaccensis* and *zebrina*, are hereby most common, confirming their significant contribution to cultivar genomes (Carreel et al., 1994; Perrier et al., 2009; Christelová et al., 2017). In contrast, translocations 1/9, 2/8, and 7/8, associated with *burmannica*, are rare, reflecting the limited influence of this genomic background (Carreel et al., 1994; Perrier et al., 2011). The 1/7 translocation, present in many cultivars, points to a yet-unknown *Musa* gene pool (Martin et al., 2020). Shepherd (1999) observed that banana cultivars with a single translocation show moderate to high gametophytic fertility, whereas those with multiple complexes exhibited reduced fertility. Pairing and recombination during meiosis normally only occurs between homologous chromosomes, but when translocations are present, recombination can also occur between non-homologous chromosomes. As these crossovers cannot be resolved they impair chromosome segregation, which results in unviable gamete formation (Yuan et al., 2021).

Next to reduced male and female fertility, diploid (2n) gamete formation is another factor linked to meiotic instability in *Musa*. Sexual polyploidization in the domestication process of banana underlies the large existing diversity of triploid and tetraploid cultivars. An example is the ‘Mchare’ diploid cultivar group which has been identified as the 2n gamete donor for five different triploid cultivars, including the widely known ‘Cavendish’ banana (Raboin et al., 2005; Hippolyte et al., 2012; Martin et al., 2023a). In plants, 2n pollen grains are approximately 25% larger in diameter than haploid pollen, due to an increase in both nuclear and cytoplasmic volume relative to chromosome number (Darlington, 1937; Bretagnolle and Thompson, 1995; Tenkouano et al., 1998). With pollen diameters in fertile wild diploid bananas ranging from 85 to 128 µm, previous research assumed this to be the size range of haploid pollen, and classified pollen between 140 and 160 µm diameter as diploid, while pollen with diameters exceeding 180 µm were considered tetraploid (Sathiamoorthy, 1994; Ortiz, 1997). However, the relation between these pollen sizes and the ploidy level of the pollen have not been experimentally determined yet in banana. In wild AA and BB genotypes, no larger pollen were encountered, whereas among the other genomic groups the frequency at which larger pollen producing genotypes were found varied: 22% of assessed AAcv genotypes produced larger pollen, 56% of AAA, 17% of AAB, 14% of ABB, and 5-25% of EAHBs (Ortiz, 1997; Ssebuliba et al., 2008). Segregation studies in diploid *Musa* suggest a genetic basis for 2n pollen production in hybrids with a dominant allele promoting their formation (Ortiz, 1997). This allele is likely more common in triploid AAA bananas, due to unilateral sexual polyploidization from diploid parents producing both 2n and n gametes (Sathiamoorthy, 1994; Ortiz, 1997; Ssebuliba et al., 2008).

Our aim was to quantify the prevalence of meiotic irregularities among a diverse panel of banana genomic groups, in order to determine their role in both aneuploid and polyploid gamete formation. Aneuploid gametes lead to reduced fertility and it is therefore of importance to determine which mechanisms underlie their formation in the context of improving banana breeding. Polyploid gametes reflect spontaneous sexual polyploidization, which is key in the formation of current triploid landraces, and may be leveraged in current banana triploid breeding. We studied these processes on the male side as it is much more accessible than female meiosis. We also aim to determine whether pollen size can be used as an indication for ploidy in banana. Moreover, we aim to determine how structural heterozygosity, resulting from complex ancestries and chromosomal translocations in cultivated bananas, affects male meiotic stability.

## Materials and methods

### Plant materials

Two types of plant material were collected, i.e., fully developed anthers at anthesis containing mature pollen for pollen size and nuclear analysis, and immature male flower clusters to study meiosis.

Mature pollen samples were obtained from the International Institute of Tropical Agriculture (IITA) in Arusha, Tanzania and from the Brazilian Agricultural Research Corporation (Embrapa) located in Bahia, Brazil. In Tanzaniam male flower clusters at anthesis were collected from 70 different *M. acuminata* wild (AA) and cultivated (AAcv) genotypes, and one *M. balbisiana* genotype (BB). For most genotypes, flowers were collected from three separate (but clonal) plants, unless only two or one flowering plants were available. An overview of the genotypes sampled and the amount of biological repeats obtained is given in **Supplementary Table S1**. Anthers were immediately dissected from the sampled flower clusters, after which half of the anthers was immediately fixed with Clarke’s solution (75% ethanol, 25% acetic acid) to preserve the nuclear integrity of the pollen. The other half was air-dried for three days at room temperature (20 °C) to allow preservation of pollen for high-throughput pollen size assessment. Both types of anther samples were then transported to Heverlee, Belgium, for microscopy analysis. For a more diverse panel of 31 genotypes including AA, AAcv, BB, AAA, AAB, ABB and AAAB genotypes, clusters of male flowers at anthesis were sampled in the field in Brazil. An overview of the sampled genotypes is given in **Supplementary Table S2**. Anthers were separated from the remaining floral structures and air-dried for three days at 25 °C room temperature before shipment to Belgium. No anthers were preserved in Clarke’s fixative due to logistical constraints.

Entire male flower buds were collected from a broad range of different Musa genotypes, including AA, AAcv, BB, AAA, ABB, AAAA and AAAB genotypes. These samples were obtained from the research fields of Sendusu (Uganda, IITA), Arusha (Tanzania, IITA), Bahia (Brazil, Embrapa), as well as from greenhouses in Meise (Belgium, Meise Botanic Garden) and Heverlee (Belgium, KU Leuven). An overview of the sampled genotypes, their location, and the date of sampling is given in **Supplementary Table S3**. Male flower buds were dissected by sequentially removing spathes and their associated flower clusters until either the bud was fully depleted or the flowers were smaller than 3 mm in length, corresponding to meiotic stage flowers. The outermost hand of flowers in anthesis was designated as stage 0, with each younger hand numbered incrementally (e.g., stage 1, stage 2, etc.). From stage 15 onwards, entire flowers were fixated for all subsequent stages, i.e. up to stages 30–40 depending on the genotype. Flower tissues were placed into individual 1.5 mL tubes and were fully submerged in Clarke’s fixative and stored overnight at 4 °C. After 24 hours, the fixative was replaced by 70% ethanol solution and samples were further stored at 4 °C until processing.

### High throughput imaging-based pollen size analysis

To extract pollen from dried anther samples, three anthers per sample were placed in a 1.5 mL microcentrifuge tube containing 1 mL of pollen buffer and vortexed for 1 minute to release the pollen. The buffer consisted of 0.5 M EDTA, 1.6 mM H_3_BO_3_, 17% (w/v) sucrose, and 0.1% (v/v) Triton X-100, adjusted to a pH of 7.5, and facilitated pollen release and minimized pollen clumping (Lim et al., 2020). Following release of the pollen from the anther locules the remaining anther pieces were discarded and the pollen suspension was centrifuged at 500 rpm for 2 minutes. The supernatant was removed, and the pellet was resuspended in 1 mL fresh pollen buffer, followed by a second round of centrifugation under the same conditions. The resulting pellet was then resuspended in a mixture of 300 µl 9% sucrose solution and 300 µl pollen buffer. From this suspension, six times 50 µl was manually pipetted into six wells of a Greiner 96-well plate. Imaging was performed using a LEICA DMi8 inverted microscope with K5 camera, via automated acquisition settings. Brightfield composite images of the centers of individual wells were captured at 200× magnification by stitching overlapping fields (15% overlap).

Pollen images were analyzed using CellProfiler software (version 4.2.4) (Lamprecht et al., 2007) using a custom image analysis pipeline to correctly identify pollen grains and measure parameters related to their shapes and sizes. Erroneously identified objects, i.e., selected objects that did not correspond to pollen grains, were removed from the dataset to retain data of actual pollen grains only. Based on nuclear staining, an equivalent diameter of 120 µm was used as a lower threshold to distinguish putative higher ploidy pollen from haploid pollen, and an equivalent diameter of 60 µm as an upper threshold for aneuploid pollen. The indirectly estimated percentage of higher ploidy pollen within each sample was then calculated by dividing the amount of pollen with diameters above 120 µm by the total amount of measured pollen grains and multiplying by 100. Similarly, to estimate the percentage of aneuploid pollen the amount of pollen with diameters below 60 µm was divided by the total amount of pollen and multiplied by 100.

### Pollen nuclei size measurements

Staining and subsequent imaging of nuclei in fixated *Musa* pollen grains was performed according to a protocol of Huang et al. (2013) with minor adaptations. Epi-fluorescent imaging was performed using a LEICA DMi8 inverted microscope equipped with K5 camera in two fluorescence channels: the TXR channel (540–580 nm) to capture the PI staining of the nuclei, and the LED405 channel (375–435 nm) to record pollen autofluorescence. During image processing in CellProfiler, background fluorescence from the LED405 channel was subtracted from the TXR signal to trim the PI signal and enhance nuclear visibility relative to the cytoplasm. Subsequently, ImageJ (Rasband, 2018) was used to manually measure the area of the generative pollen nuclei, as well as the equivalent diameter of the corresponding pollen grains.

### Male meiotic tetrad visualization and counting

Meiotic end products in Musa spp. were visualized through lactopropionic orcein (2%) staining of tetrad stage anthers of fixated flowers (Ross et al., 1996). As *Musa* male meiosis undergoes successive cytokinesis and therefore meiosis II (MII) meiocytes might be confused with restituted dyads, accurate stage selection was critical for reliable tetrad analysis. When multiple consecutive flower stages contained tetrad-stage meiocytes, only the flower stage closest to microspore dissociation was analyzed to ensure no dividing meiocytes were present. The flower stage corresponding to the male meiotic tetrad stage is given in **Supplementary Table S3** for each analyzed inflorescence. Slides were examined visually under a Nikon TE300 light microscope at 200× magnification. Per flower, a minimum of 200 male meiotic products were counted and categorized based on shape and the number of compartments (**Figure 1**).

**Figure 1:**
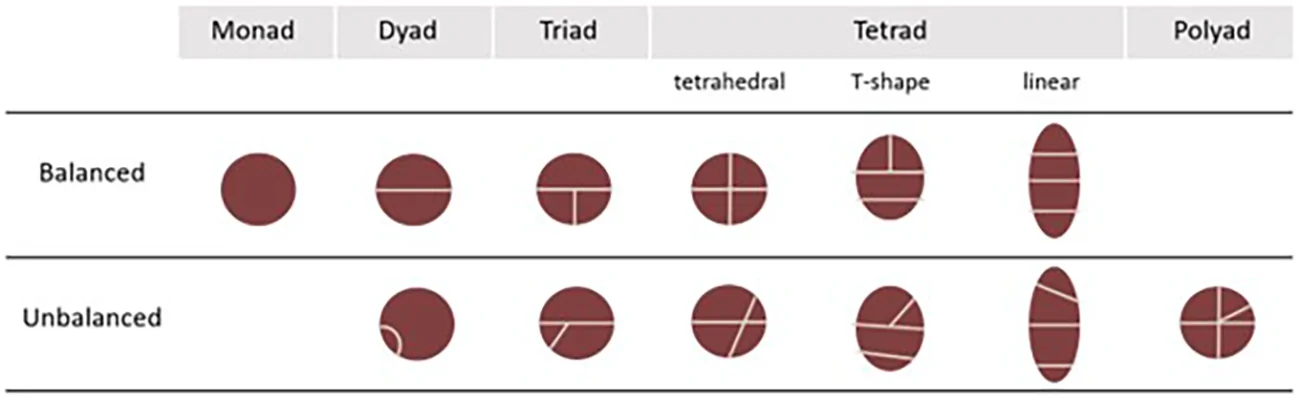
Classification of meiotic end products.

### Male meiotic chromosome spreading

Male meiotic chromosome spreads were prepared according to the procedure of Ross et al. (1996). To ensure capturing all stages of meiosis, ranging from early leptotene to telophase II, the three meiotic flower stages immediately preceding the tetrad stage were consistently used for preparing chromosome spreads. For each selected flower stage, three ~1 mm segments were excised from three separate anthers from three separate fixated flowers. Prepared slides were examined under a Leica DMi8 fluorescence microscope (Leica Microsystems) at 1000× total magnification. DAPI-stained chromosomes were visualized using a K5 camera.

### Statistical data analysis

All statistical analyses were performed in Rstudio using R v. 4.0.5 (R Studio Team, 2021). The proportions of meiotic products observed during the tetrad stage were compared separately between the genomic groups and the genotypes under study via generalized linear mixed models with a binomial error distribution and a logit link function that transforms probabilities into a linear scale. The models were fit using the *lme4* package in R (Bates et al., 2015). The AA group was used as a reference genomic group and ‘Borneo’ was used as a reference genotype. To assess model fits, marginal and conditional R² were computed using the *r.squaredGLMM()* function from the *MuMIn* package (Bartoń, 2025).

In order to detect putative location effects on pollen diameter for seven genotypes that were common between the dried pollen samples from Tanzania, and Brazil, the *lme4* package was used to construct a linear mixed-effects model. Model assumptions (normality of residuals and homogeneity of variance) were evaluated using residual plots and the Shapiro-Wilk test. Additionally, Kruskal-Wallis tests were performed to determine the effect of location on pollen diameter for the separate genotypes. To detect differences in mean pollen diameter, amount of pollen measured per flower, the percentage of large pollen (pollen diameter > 120 µm) and the percentage of small pollen (pollen diameter < 60 µm) between different genome groups (AA, BB, AAcv, AAA, ABB and AAAB), Kruskal-Wallis tests and *post-hoc* Wilcoxon tests were performed in Rstudio. This was done separately for the pollen samples obtained from Tanzania and the ones obtained from Brazil.

We also assessed the influence of meiotic instability on mature pollen size. To this end, the percentages of small and large pollen of the accessions for which also a tetrad stage analysis was performed, were modelled separately over the different meiotic product types via a linear model using the *lm()* function. Finally, we aimed to discern the effects of ploidy, complex ancestries and structural heterozygosity in cultivated bananas on the stability of meiosis, pollen amount and mature pollen size via a linear model using the *lm()* function. Data on the amount of ancestors and percentage of heterozygosity was obtained from Martin et al. (2023b), while data on translocations was obtained from Martin et al. (2020). The data and genotypes used for this are presented in **Supplementary Table S4**. For genotypes for which pollen size data was available from both Tanzania and Brazil sites, average values over both locations were used for both the percentages of small and large pollen.

## Results

### Characterization of meiotic stability in *Musa* spp.

Stability of male meiosis was assessed in 31 AA, AAcv, BB, AAA, ABB, AAAA and AAAB genotypes through an analysis of the meiotic products found at the tetrad stage. The mean percentages and standard errors of balanced and unbalanced tetrads, restituted products (monad, dyads and triads), and polyads for the individual genotypes are shown in **Figure 2**. The majority of tetrads in all genotypes was balanced (**Figure 2A**). Within both unbalanced and balanced groups, 80.3% tetrads had the tetrahedral configuration, followed by T-shape with 11.4%, whereas linear configurations appeared least frequently with 8.3% (**Figures 2F–H**). Balanced tetrads are the outcome of an undisturbed meiotic process and their frequency therefore represents the stability of meiosis. The AA genome group that was used as a reference displayed 96.9 ± 0.6% balanced tetrads. Among the other groups, AAcv, BB and AAAB, showed similar values with 94.6 ± 1.2%, 85.3 ± 12.2% and 83.5 ± 18.7% balanced tetrads respectively. AAAA, ABB and AAA groups displayed significantly lower frequencies of balanced tetrads at 71.2 ± 14.0% (*z* = -3.0, *p* = 0.002), 66.4 ± 21.5% (*z* = -2.4, *p* = 0.01) and 49.8 ± 15.2% (*z* = -4.659, *p* < 0.0001) respectively. Genotypes with a percentage of balanced tetrads significantly lower than the ‘Borneo’ AA reference are indicated in **Figure 2A**. According to expectations, the diploid genome groups showed the highest meiotic stability, followed by tetraploid genome groups and the least stable meiosis was displayed by triploid groups. Hybrids between two species usually result in decreased meiotic stability due to mismatches between divergent chromosomes. This effect was not noticeable here however, with the AAAB and ABB group showing higher balanced tetrad frequencies than the AAAA and AAA groups. Unbalanced tetrads are typically formed due to unbalanced chromosome segregation during meiosis and result in aneuploid microspores (Dodds and Simmonds, 1946; Shepherd, 1999). The frequency of unbalanced tetrads was however quite low among the different groups, with the AA reference group displaying only 1.1 ± 0.4% unbalanced tetrads, and AAcv, BB, ABB and AAAB showing very similar percentages. Though not significantly different from the AA reference, the AAAA genome group showed 5.7 ± 4.7% unbalanced tetrads (*z* = 1.8, *p* = 0.07). Only the AAA group produced a significant amount of unbalanced tetrads with 7.5 ± 5.3% (*z* = 2.4, *p* = 0.02). Genotypes with a percentage of unbalanced tetrads significantly higher than the ‘Borneo’ AA reference are indicated in **Figure 2A**.

**Figure 2:**
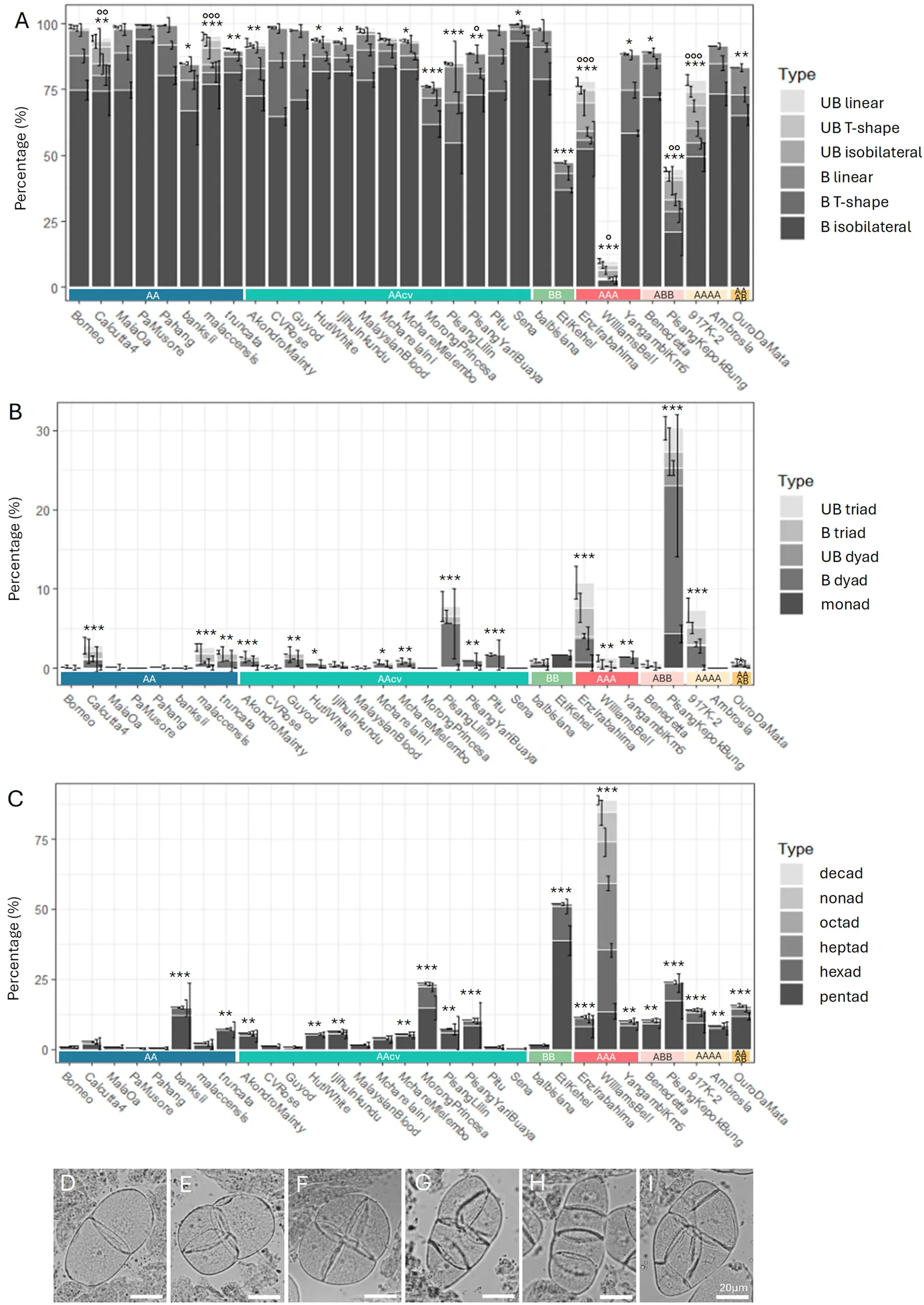
Evaluation of the stability of male meiosis for different *Musa* genotypes within the AA, AAcv, BB, AAA, ABB, AAAA and AAAB genome groups. ‘B’ stands for balanced meiotic products, ‘UB’ for unbalanced meiotic products. **(A)** Percentage of linear, T-shaped and tetrahedral tetrads, with ‘*’ indicating significantly lower values of balanced tetrads compared to the ‘Borneo’ reference, and ‘°’ indicating significantly higher values of unbalanced tetrads compared to ‘Borneo’. **(B)** Percentage of restituted products: monads, dyads and triads, with ‘*’ indicating significantly higher values compared to ‘Borneo’. **(C)** Percentage of polyads, ranging from pentads (with five compartments) up to decads (ten compartments), with ‘*’ indicating significantly higher values compared to ‘Borneo’. **(D–I)** Representative bright field images of different types of male meiotic products in ‘Pisang Lilin’, including a dyad **(D)**, triad **(E)**, tetrahedral tetrad **(F)**, T-shape tetrad **(G)**, linear tetrad **(H)**, and a polyad **(I)**. Scale bar; 20 µm. *' indicates p<0.05, '**' indicates p<0.01 and '***' indicates p<0.001.

Whereas unbalanced tetrads thus account for only a small part of aberrant meiotic products in *Musa* spp., polyads account for the majority (**Figure 2I**), as is also noticeable from the strong negative correlation they display towards the presence of tetrads (R = -0.95, *p* < 0.0001) (**Supplementary Figure S1C**). Polyads stem from defects in chromosome segregation that lead to lagging or fragmented chromosomes and subsequently the formation of micronuclei (Shin et al., 2021). The resulting microspores are aneuploid. The AA reference group produced only 1.4 ± 0.4% polyads, and the AAcv group produced a similar 2.8 ± 0.7% polyads. The remaining groups all showed significantly higher polyad percentages, except for AAAB where the difference was not significant due to the large variation in polyad formation in the different flowers of the examined inflorescence. Polyad formation accounted for 12.1 ± 10.4% of meiotic products in BB (*z* = 2.2, *p* = 0.03), 12.5 ± 7.4% in AAAA (*z* = 3.1, *p* = 0.002), 15.7 ± 17.9% in AAAB (*z* = 1.9, *p* = 0.06), 17.2 ± 13.6% in ABB (*z* = 2.7, *p* = 0.008) and 21.5 ± 10.2% in AAA (*z* = 4.4, *p* < 0.0001). Genotypes with a percentage of polyads significantly higher than the ‘Borneo’ AA reference are indicated in **Figure 2C**. High percentages of polyads were expected in the triploid genome groups due to the unbalanced segregation of their uneven chromosome number. In tetraploids, which have an even chromosome number, meiosis can in theory progress normally, but clearly meiotic aberrations are present in the tetraploids under study that lead to substantial polyad formation. Also, the large amount of polyads in the wild BB group, in which meiosis is expected to occur error-free, was striking. Whereas one of the two examined BB genotypes showed the expected high frequency of balanced tetrads, i.e. ‘balbisiana’ with 97.6 ± 1.9%, ‘Eti kehel’ showed only 47.5 ± 19.2% balanced tetrads and 51.6 ± 18.5% polyads, indicating the occurrence of meiotic aberrations in this genotype. The current set-up does not allow us to determine if this is representative of the BB genome group or due to environmental effects. Other genotypes showing polyad levels that exceed the base level of polyad formation of their genome group are ‘banksii’ with 8.8 ± 4.3% polyads followed by ‘truncata’ with 7.1 ± 3.5% in the AA group, ‘Morong Princesa’ with 23.5 ± 13.3% followed by ‘Pisang Jari Buaya’ with 7.5 ± 3.1% in the AAcv group, ‘Pisang Kepok Bung’ with 27.3 ± 149% in the ABB group, and especially ‘Williams Bell’ in the AAA group with 88.7 ± 7.5% polyads. For most genotypes, the main polyad type were pentads, indicating a rather low level of aneuploidy. In ‘Williams Bell’, however, meiocytes with up to ten compartments were observed, coinciding with severe aneuploidy in resulting microspores.

Finally, another small part of aberrant meiotic products were the direct consequence of meiotic restitution, namely monads, (un)balanced dyads and (un)balanced triads, which result in the formation of 2n and 4n gametes (**Figures 2D, E**). Among these, 50.2% were balanced dyads, followed by 24.6% unbalanced dyads and 20.8% unbalanced triads. The AA reference group showed only 0.4 ± 0.4% restituted products, similar to what was observed in the AAAB. In the same range but significantly higher are the values observed in AAcv with 0.7± 0.2 (*z* = 2.1, *p* = 0.04), BB with 0.6 ± 0.8 (*z* = 3.0, *p* = 0.002) and AAAA with 2.5 ± 2.2% (*z* = 2.0, *p* = 0.046). Higher values were observed in the ABB group with 4.4 ± 5.2% (z = 1.3, p = 0.18) and the AAA group with 4.6 ± 3.4% (*z* = 0.4, *p* = 0.7), but these were not significantly different from the control due to the large variation in the trait. Genotypes with a percentage of meiotic restitution products significantly higher than the ‘Borneo’ AA reference are indicated in **Figure 2B**. Whereas a baseline production of up to 2% meiotic restitution products was relatively frequently observed in the genotypes, only a few formed substantial amounts of restituted products, including ‘Pisang Lilin’ of the AAcv group with 7.1 ± 1.7%, Enzirabahima of the AAA group with 10.1 ± 3.2%, Pisang Kepok Bung of the ABB group with 28.6 ± 12.6% and 917K-2 of the AAAA group with 7.2 ± 2.4%. No correlation existed between dyad and polyad formation (**Figure 2A**). Overall, besides a base level of polyad formation and meiotic restitution in most genotypes, only certain genotypes produce high frequencies of polyads or restituted products. As these are not essentially co-occurring, this indicates that they might be triggered by different and genotype-specific genetic aspects.

Besides evaluating the stability of male meiosis, we also assessed meiocyte size as an indication for premeiotic doubling. Significantly enlarged tetrads or other types of meiotic products were not observed in any of the assessed genotypes, except for ‘Pisang Lilin’. In its tetrad stage, 2 out of 117 (1.7%) assessed male meiocytes were found to be considerably larger, with a Feret diameter of 72.76 ± 1.03 µm compared to 50.50 ± 6.35µm (**Supplementary Figure S2**). This indicates the presence of higher ploidy meiocytes, most likely resulting from premeiotic genome doubling.

Via linear regression models we investigated to what extent the prevalence of polyads and meiotic restitution products are determined by structural genomic features inherent to the genotypes, including ploidy level, percentage heterozygosity, the amount of ancestor genome contributions and the amount of translocations. The genotypes and all data obtained from previous publications used in this analysis are listed in **Supplementary Table S4**. The model fit for the percentage of polyads was able to explain almost 40% of the observed variation in (Adj. R² = 0.39), but this was solely due to the significant positive effect of ploidy (*β* = 41.8, *SE* = 18.1, *p* = 0.04), confirming that more polyads are formed and the instability of meiosis increases when ploidy increases. The amount of ancestor contributions, translocation and the heterozygosity of the genotypes did not show any significant effects on polyad formation. The same model was unable to explain any of the variation in the percentage of meiotic restitution products (Adj. R² = -0.16), with none of the tested parameters having a significant effect. This further indicates that the occurrence of meiotic restitution might be more related to certain genetic factors instead of genomic structure.

### AA and BB reference meiotic chromosome dynamics

To assess the normal course of MI and MII chromosome dynamics in *Musa* male meiosis, chromosome spreads were prepared from *M. acuminata* ssp. *microcarpa* ‘Borneo’ and *M. balbisiana* ‘balbisiana’ that showed stable meiotic outcomes consisting of 97-98% balanced tetrads (**Figures 3A–F**; **Supplementary Table S5**). During prophase I, progressive chromosome condensation commences at the leptotene stage. Chromosome pairing starts during zygotene and is followed by the establishment of the synaptonemal complex between homologs at the pachytene stage (**Figures 3A–D**). At this stage, meiotic recombination occurs, linking homologous chromosomes together as bivalents. Overall, no major synapsis defects were observed. As meiosis progresses through diplotene, the synaptonemal complex disassembles and bivalents further condense until at diakinesis. At this stage, 11 highly condensed bivalents were consistently observed (**Figures 3E–H**), aligning with the expected number of bivalent for diploid *Musa* genotypes (2n = 2x = 22). In *M. balbisiana*, a significant fraction of diakinesis-stage meiocytes (27.3%; n = 11) hereby displayed ectopic clustering of three bivalents (**Figures 3G, H**). At metaphase I, the bivalents consistently aligned along the equatorial plane, showing the same orientation towards the poles of the cells which indicates their attachment to the meiotic spindle, even when a bivalent is separate from the remaining bivalents (**Figures 3I–L**), after which homologous chromosomes segregated during anaphase I (**Figures 3M–P**), moving towards opposite poles at telophase I. During interkinesis, the two resulting chromosome sets (n = 11) are partially decondensed and separated by a cell wall following the first cytokinesis (**Figures 3Q–T**).

**Figure 3:**
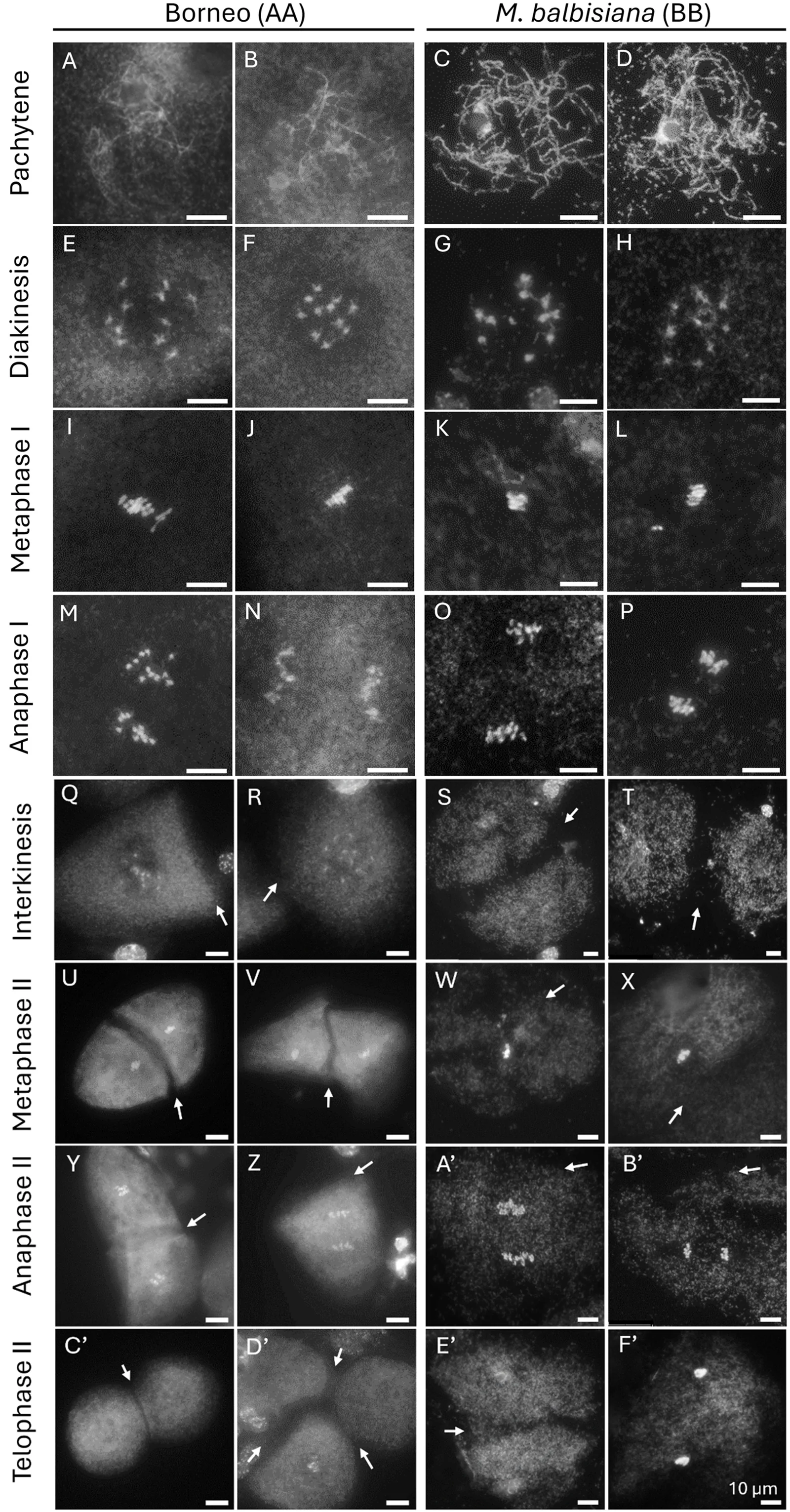
Chromosome dynamics throughout the different stages of male meiosis I and II in *M. acuminata* ssp. *microcarpa* ‘Borneo’ and *M. balbisian*a as visualized through DAPI-stained chromosome spreading. **(A–D)** Pachytene; **(E–H)** Diakinesis; **(I–L)** Metaphase I; **(M–P)** Anaphase I; **(Q–T)** Interkinesis **(U–X)** Metaphase II; **(Y–B’)** Anaphase II; **(C’–F’)** Telophase II. White arrows indicate division planes. Scale bars are at 10 µm.

Subsequently, during metaphase II (**Figures 3U–X**), chromosome sets in both daughter cells aligned along the corresponding metaphase plate, after which sister chromatids segregated during anaphase II (**Figures 3Y–B’**). Notably, 11.8% (n = 17) of anaphase II meiocytes examined in ‘Borneo’ showed lagging chromosomes (**Supplementary Table S5**). Finally, male meiosis in both genotypes concluded with re-nucleation and a second cytokinesis, yielding tetrad-stage meiocytes that contain four separated haploid chromosome sets.

### Meiotic defects underlying polyad formation in wild AA and BB genotypes

Though belonging to the group of wild diploids, ‘banksii’ (AA) and ‘Eti Kehel’ (BB) were found to produce significant amounts of polyads, indicating defects in meiotic chromosome segregation. In order to find the underlying cytological mechanisms, male meiotic chromosome dynamics were evaluated in these genotypes (**Figures 4A–P**; **Supplementary Table S5**).

**Figure 4:**
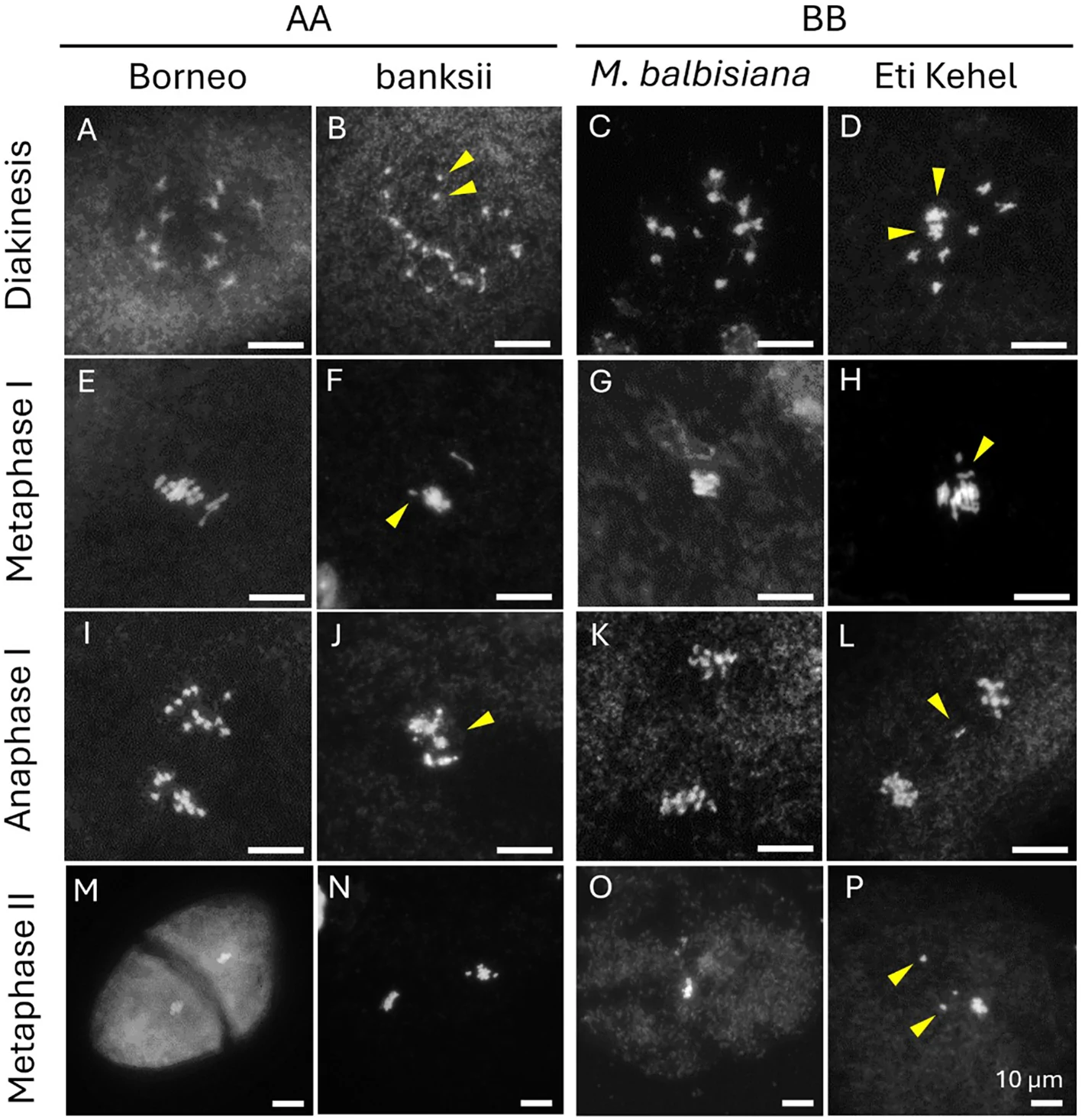
Representative images of chromosome dynamics at different stages in male meiosis of the reference *Musa* genotypes ‘Borneo’ (AA) and *M. balbisiana* (BB), as well as cytological aberrations observed in male meiosis of ‘banksii’ (AA) and ‘Eti Kehel’ (BB). **(A–D)** Diakinesis; **(E–H)** Metaphase I; **(I–L)** Anaphase I; **(M–P)** Metaphase II. Arrow heads indicate univalents **(B, F, H)**, bivalent clusters **(D)**, and lagging chromosomes **(J, L, P)**. White arrows indicate division planes. Scale bars are 10 µm.

At diakinesis, ‘Borneo’ exhibited only cells with 11 distinct bivalents, while among the seven observed ‘banksii’ diakinesis cells, two displayed alterations in bivalent formation (28,6%) with one containing cluster of three bivalents in approximately a quarter of diakinesis-stage meiocytes. Similarly, in ‘Eti Kehel’ 30.8% (n = 13) of cells showed clusters of three bivalents (**Figure 4D**). At metaphase I, one out of six observed meiocytes in ‘banksii’ displayed a univalent (16.7%) (**Figure 4F**) and in ‘Eti Kehel’, 10.3% (n = 29) of cells showed univalents (**Figure 4H**) and another 3.4% displayed ectopic non-homologous pairing. At anaphase I, 60.0% (n = 5) of ‘banksii’ cells showed lagging bivalents (**Figure 4J**). Also, in ‘Eti Kehel’ many defects were observed during anaphase I, which were not visible in the *M. balbisiana* reference, with 54.5% (n = 11) of cells showing lagging bivalents (**Figure 4L**) and another 27.3% (n = 11) exhibiting anaphase bridges (**Supplementary Figure S3A**). The latter defect was still noticeable during interkinesis in which 8.3% (n = 12) of ‘Eti Kehel’ meiocyte cells displayed chromosome bridges (**Supplementary Figure S3B**). Among four observed metaphase II cells in ‘Eti Kehel’ one exhibited clear defects in the orientation of the metaphase plate (**Figure 4P**), whereas this was not observed in the *M. balbisiana* reference. At telophase II, ‘Eti Kehel’ meiocytes showed balanced segregated chromosome sets, indicating normal reductional division and regular separation of chromosomes. However, taking into account that only two cells were observed at this stage, defects observed in earlier stages are probably not reflected due to the small number of observations.

Both ‘banksii’ and ‘Eti Kehel’ thus display higher frequencies of meiotic abnormalities as compared to the reference AA and BB lines, with particular the presence of univalents in diakinesis and metaphase I as well as lagging bivalents in anaphase I. In ‘Eti Kehel’, chromosome bridges were observed during anaphase I that persisted up to telophase I, possibly explaining the increased severity of polyad formation over ‘banksii’.

### Meiotic defects underlying polyad formation and meiotic restitution in AAcv genotypes

Tetrad analysis revealed that some AAcv genotypes exhibit increased frequencies of polyad formation with particularly ‘Pisang Lilin’ and ‘Pisang Jari Buaya’ showing significant alterations in meiotic cell division as reflected by the ectopic formation of polyads and/or restituted products. Meiotic chromosome behavior in the AAcv genotypes ‘Pisang Lilin’ and ‘Pisang Jari Buaya’ is generally more unstable compared to AA ‘Borneo’ and ‘banksii’ genotypes, with defects in nearly all meiotic stages (**Figures 5A–N’**; **Supplementary Table S6**).

**Figure 5:**
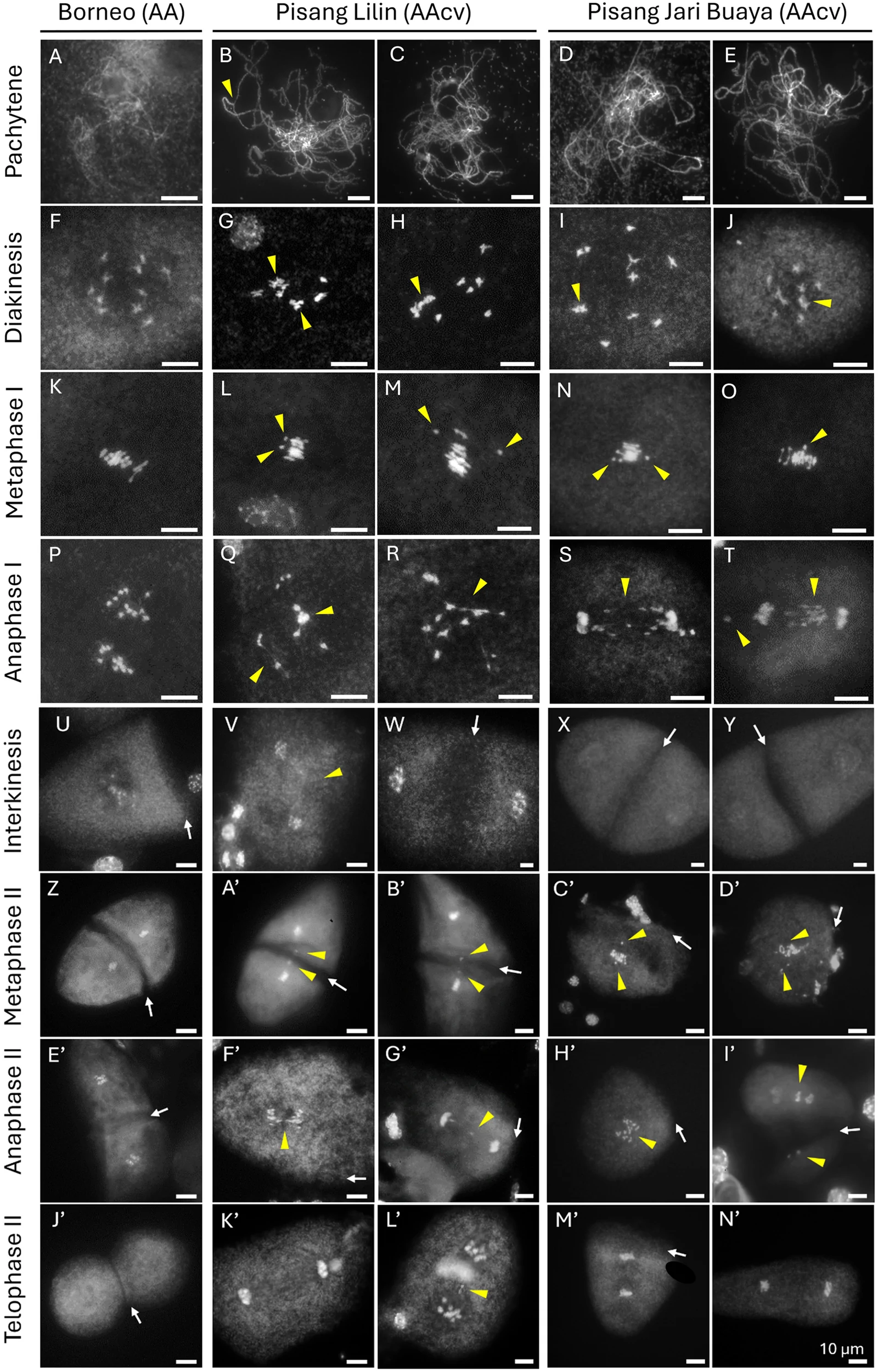
Meiotic chromosome spreads of AA reference ‘Borneo’ and representative images of cytological aberrations observed in male meiosis of AAcv ‘Pisang Lilin’ and ‘Pisang Jari Buaya’. **(A–E)** Pachytene; **(F–J)** Diakinesis; **(K–O)** Metaphase I; **(P–T)** Anaphase I; **(U–Y)** Interkinesis; **(Z–D’)** Metaphase II; **(E’–I’)** Anaphase II; **(J’–N’)** Telophase II. Arrow heads indicate **(B)** asynapsis, **(G–J)** bivalent clusters, **(L–O)** univalents, **(Q, R)** anaphase bridges, **(Q, S ,T, F’–I’, L’)** lagging chromosomes **(V)** lack of cytokinesis, **(A’, B’)** chromosome laggards at division plane, and **(C’, D’)** premature separation of chromatids. White arrows indicate division planes. Scale bars are at 10 µm.

In ‘Pisang Lilin’, 21.6% (n = 37) of pachytene cells exhibit large regions of asynapsis (**Figure 5B**). In ‘Pisang Jari Buaya’ all pachytene-stage cells appeared normal, with a full pairing and synapsis of homolog chromosomes. During diakinesis, ‘Pisang Lilin’ showed bivalent clusters in 56.5% (n = 23) of observed cells (**Figures 5G, H**), whereas in ‘Pisang Jari Buaya’ only 11.5% (n = 26) of cells displayed bivalent clusters (**Figures 5I, J**). Moreover, a fraction of ‘Pisang Jari Buaya’ diakinesis meiocytes (7.7%; n=26) showed two univalents, indicating partial loss of obligate cross-over (CO) formation. Similarly, in ‘Pisang Lilin’, a part of observed metaphase I meiocytes (9.0%, n = 78) displays two to four univalents (**Figures 5L, M**), however, notwithstanding this defect, most metaphase I cells progressed normal with a proper alignment of all chromosomes along the equatorial zone. In metaphase I cells of ‘Pisang Jari Buaya’ several defects were observed; including univalents in 28.1% (n = 32) of cells (**Figures 5N, O**), non-homologous recombination in 3.1% (**Supplementary Figure S4B**), and lack of metaphase plate orientation in another 3.1% of cells (**Supplementary Figure S4B**). In anaphase I, ‘Pisang Lilin’ showed lagging bivalents in 25.0% of cells (n = 20), as well as anaphase bridges in 5.0% of cells (**Figures 5Q, R**). Similarly, at this stage, ‘Pisang Jari Buaya’ showed a severe decline in meiotic stability with only 21.4% (n = 14) of anaphase I cells showing regular, balanced segregation of homologous chromosomes. The other cells either exhibited lagging bivalents (28.6%), anaphase bridges (35.7%) or univalents (14.3%) (**Figures 5S, T**). At telophase I, all observed cells appeared to have normally divided in both AAcv genotypes, but the number of observations was too limited to make general conclusions about stability of MI. In ‘Pisang Jari Buaya’, the observed cells also appeared normal at interkinesis with equally sized nuclei in both daughter cells (**Figures 5X, Y**). ‘Pisang Lilin’, however, had 20.0% (n = 10) of meiocytes displaying a complete lack of cytokinesis (**Figure 5V**). Metaphase II meiocytes in ‘Pisang Lilin’ showed only minor defects with laggards close to the dividing plane in 7.9% (n = 40) of cells (**Figures 5A, B’**). Similarly, among the observed metaphase II ‘Pisang Jari Buaya’ meiocytes, 18.8% (n = 16) showed premature separation of chromatids (**Figures 5C, D’**) and 12.5% (n = 16) lacked proper metaphase plate orientation. During anaphase II, meiosis seemed severely distorted in ‘Pisang Lilin’ with 38.5% (n = 13) of meiocytes displaying anaphase bridge and another 23.1% (n = 13) showing lagging chromosomes (**Figures 5F’, G’**). Similarly, in ‘Pisang Jari Buaya’ a fraction of the meiocytes (31.3%; n = 16) also showed lagging chromosomes (**Figures 5H’, I’**). Only two meiocytes were observed at telophase II for ‘Pisang Lilin’, with one of them exhibiting a lagging chromosome (**Figure 5L’**). None of the cells observed in ‘Pisang Jari Buaya’ at this stage showed any aberrations (n=12).

### Unstable meiosis in triploids

The meiotic chromosome behavior in AAA genotypes ‘Enzirabahima’ and ‘Williams Bell’ was assessed as these genotypes display many aberrant meiotic products, with large differences between both genotypes. While ‘Enzirabahima’ shows 60.2% balanced tetrads, 10.1% restitution products and 10.8% polyads, ‘Williams Bell’ produces nearly exclusively higher order polyads at 88.7%. Male meiotic chromosome spreading revealed that both genotypes display widespread meiotic abnormalities across nearly all stages (**Figures 6A–N**; **Supplementary Table S6**).

**Figure 6:**
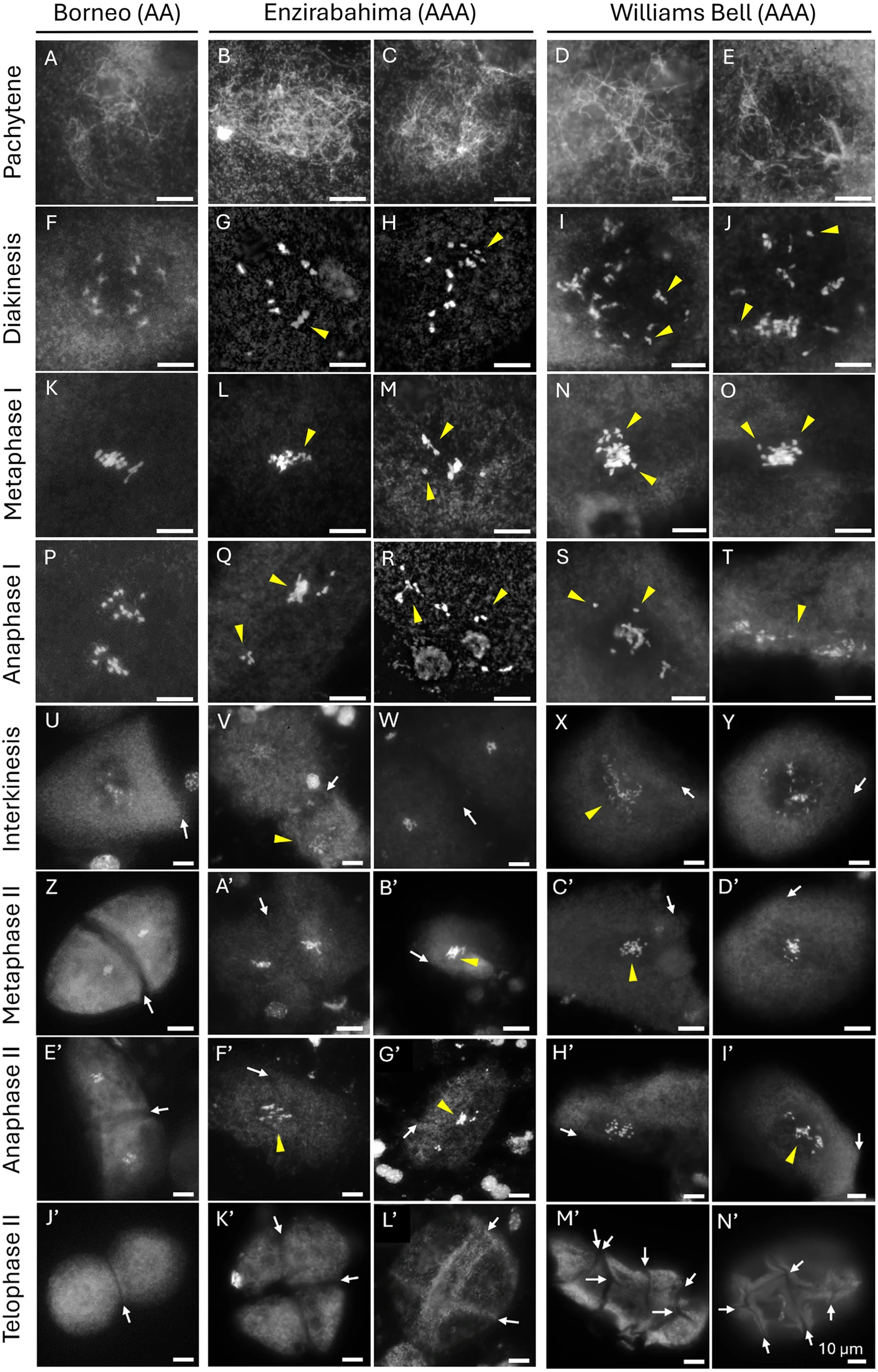
Meiotic chromosome spreads of AA reference ‘Borneo’ and representative images of cytological aberrations observed during male meiosis of AAA ‘Enzirabahima’ and ‘Williams Bell’. **(A–E)** Pachytene; **(F–J)** Diakinesis; **(K–O)** Metaphase I; **(P–T)** Anaphase I; **(U–Y)** Interkinesis; **(Z–D’)** Metaphase II; **(E’–I’)** Anaphase II; **(J’–N’)** Telophase II. Arrow heads indicate **(G)** chromosome clusters, **(H–J, M, N, O, S, X, C’)** univalents, **(L, M)** lack of metaphase plate orientation, **(R)** anaphase bridges, **(Q, T, F’, G’, I’)** lagging chromosomes **(V)** unequal division, **(M’, N’)** polyads. White arrows indicate division planes. Scale bars indicate 10 µm.

At diakinesis, ‘Enzirabahima’ showed recombination between the three homologous copies of each chromosome, forming eleven trivalents instead of eleven bivalents. One cell thereby also showed several univalents (33.3%) (**Figure 6H**), two cells exhibited clusters of three trivalents (66.7%) (**Figure 6G**). In ‘Williams Bell’, of the 18 diakinesis cells observed. 33.3% also displayed eleven trivalents, the remaining 66.7% of cells contained nearly only univalents (**Figures 6I, J**). No normal metaphase I were observed in either ‘Enzirabahima’ or ‘Williams Bell’. ‘Enzirabahima’ showed physical linkages between trivalents in all of the assessed metaphases (n = 6), putatively indicating for non-homologous recombination (**Figures 6L, M**). In contrast, ‘Williams Bell’ again showed many univalents in observed metaphase I stage cells (**Figures 6N–O**). In anaphase I, half (n = 6) of the meiocytes in ‘Enzirabahima’ exhibited lagging chromosome clusters (**Figure 6Q**), whereas the other half (n=6) displayed anaphase bridges (**Figure 6R**). In ‘Williams Bell’, only two cells in anaphase I were observed, both showing again many univalent, which lagged at the equatorial midzone of the cell in **Figure 6T**. At interkinesis, one out of seven ‘Enzirabahima’ meiocytes showed unevenly sized daughter cells, indicating that it seemed to have undergone an unequal division (**Figure 6V**). The remaining cells showed balanced segregation. In ‘Williams Bell’ only separate daughter cell compartments with decondensed chromosomes were observed, (**Figure 6X**). During metaphase II, two out of the four observed PMCs in ‘Enzirabahima’ showed chromatids that were perpendicular to the orientation of the remainder of the chromatids, indicating failure of attachment to the spindle (**Figures 6A’, B’**), whereas in ‘Williams Bell’ metaphase II in two out of the four observed cells seemed to exist only of loose chromatids with no clear polar orientation (**Figure 6C’**). In anaphase II, both genotypes showed lagging chromosomes, in two out of three observed cells in ‘Enzirabahima’ (**Figures 6F’, G’**), and in one out of three observed cells in ‘Williams Bell’ (**Figure 6I’**). Finally, at telophase II, all assessed meiocytes (n = 3) show a balanced tetrad structure in ‘Enzirabahima’, whereas ‘Williams Bell’ exhibited three higher order polyads. The main difference between both triploids is that in ‘Enzirabahima’ homologs pair and recombine, which leads to some chromosome bridges and lagging chromosomes during the meiotic cell divisions, but overall, it seems to enable chromosome segregation to occur in a relatively regular way as it results in mainly balanced tetrads, though their four compartments presumably hide unbalanced chromosome numbers. In ‘Williams Bell’ on the other hand, there was almost no recombination, resulting in many MI univalents that did not attach properly to the meiotic spindle, leading to lagging chromosomes, which can result in polyad formation when they become separated by a cell wall from the normally dividing chromosomes.

### Pollen grain characteristics: comparison between sites and genomic groups

Dried pollen samples were obtained from two locations; Arusha (Tanzania) and Bahia (Brazil). Seven genotypes were common in both samplings. Where sampling was performed under 20 °C in the dry season in Arusha, sampling in Bahia occurred under 30 °C in the rainy season. The significant location dependent variations highlight the strong influence of environment on microsporogenesis. External factors, such as temperature and humidity differences between Brazil and Tanzania, can alter the expression of key genes involved in pollen development or disrupt tapetal function during pollen wall development, potentially impacting pollen volume and viability. A linear mixed-effects model was run to assess the influence of location and genotype on pollen grain diameter (**Figure 7**). A substantial part of the variation in pollen diameter was explained by location as fixed effect, with a marginal R² of 0.29. With the random effect of genotype added, a conditional R² of 0.45 was obtained, leaving half of the observed variation unexplained. Both factors, genotype and location, were found to have a highly significant effect on pollen grain diameter (*F_5_* = 1979, *p* < 2e-16 and *F_1_* = 15308, *p* < 2e-16, respectively), with pollen grain samples from Tanzania estimated to be on average 11.5 ± 0.1 µm larger than those obtained from Brazil. Pollen diameters were indeed significantly larger in samples obtained from Tanzania for ‘Calcutta 4’, ‘Pa musore no2’, ‘Pisang Serun’, ‘Selangor’ and ‘Pisang Lilin’, though there were no significant differences for ‘Pisang Kermian’ and ‘Uwati’. Though some genotypes display similar pollen sizes between both location, taking into account the overall significant difference in pollen grain diameter between samples from Tanzania and Brazil, the pollen size datasets obtained from the two locations were analyzed separately in further pollen size analyses.

**Figure 7:**
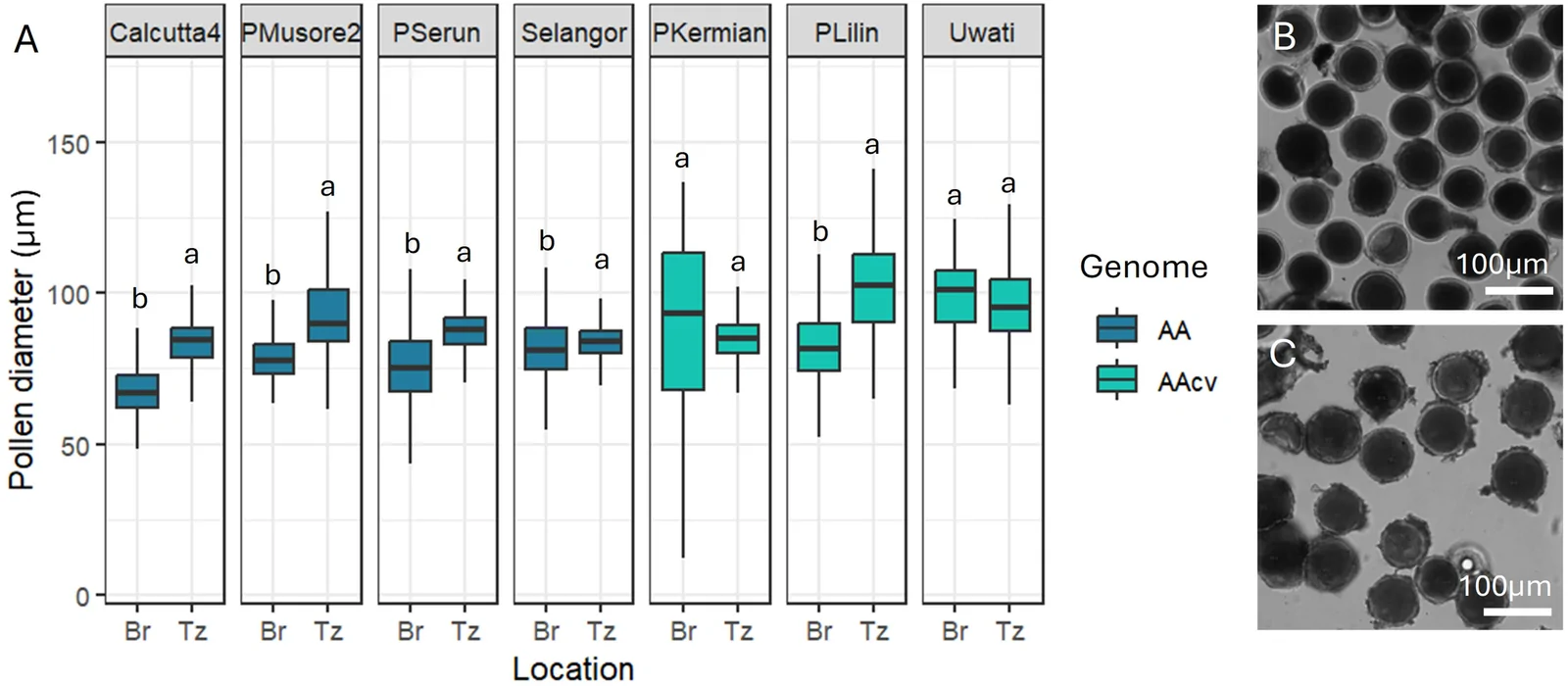
**(A)** Diameters (µm) of mature pollen grains from seven *Musa* genotypes sampled both in Brazil (Br) and Tanzania (Tz). Significant differences between locations within genotypes according to Kruskal-Wallis tests are indicated by distinct letters in the graphs. **(B, C)** Bright field image of ‘Calcutta 4’ pollen grains sampled from **(B)** Brazil and **(C)** Tanzania.

For the samples obtained from Brazil, there was an overall significant difference in pollen diameter between genome groups (*χ²6* = 20.6, *p* = 0.002) (**Figure 8A**). AA and BB groups showed pollen diameters of 72.2 ± 12.0 µm and 72.4 ± 18.1 µm, respectively. The tetraploid AAAB and triploid AAA group displayed significantly larger pollen diameters compared to the wild diploid genome groups with 99.4 ± 19.0 µm and 92.6 ± 17.4 µm respectively. In the middle with no significant differences regarding to the other genome groups, were the ABB and AAcv genome groups, with mean pollen diameter of 87.76 ± 18.1 µm and 80.60 ± 15.13 µm respectively. Also, the mean amount of pollen measured per flower showed an overall significant difference between genome groups obtained from Brazil (*χ²6* = 18.8, *p* = 0.004) (**Figure 8B**). The wild diploid BB groups displayed on average 3741 pollen grains per flower, a significantly higher pollen amount compared to AAcv, AAA, AAAB and ABB groups that respectively have 618, 251, 248 and 89 measured pollen grains per flower. Though the AA group had 3409 measured pollen grains on average, due to the large variation in pollen amounts among genotypes in this group, no significant differences with the other groups were observed. Tanzania samples were obtained only for AA and AAcv groups, overall, they followed the same trend as was seen in the data obtained from Brazil in these groups (**Supplementary Figure S5**). While mean pollen grain diameters for both genomic groups were larger as compared to those sampled in Brazil, i.e., with 87.94 µm for AA and 88.68 µm for AAcv genotypes, no significant differences were observed between both groups (*χ²2* = 0.2, *p* = 0.9). This indicates that with increases in ploidy, pollen size also increases, but the amount of pollen is also severely decreased, indicating fertility issues. Though tetraploids are expected to produce systematically (larger) 2n pollen and display higher fertility over triploids, there were no significant differences between both groups in these aspects.

**Figure 8:**
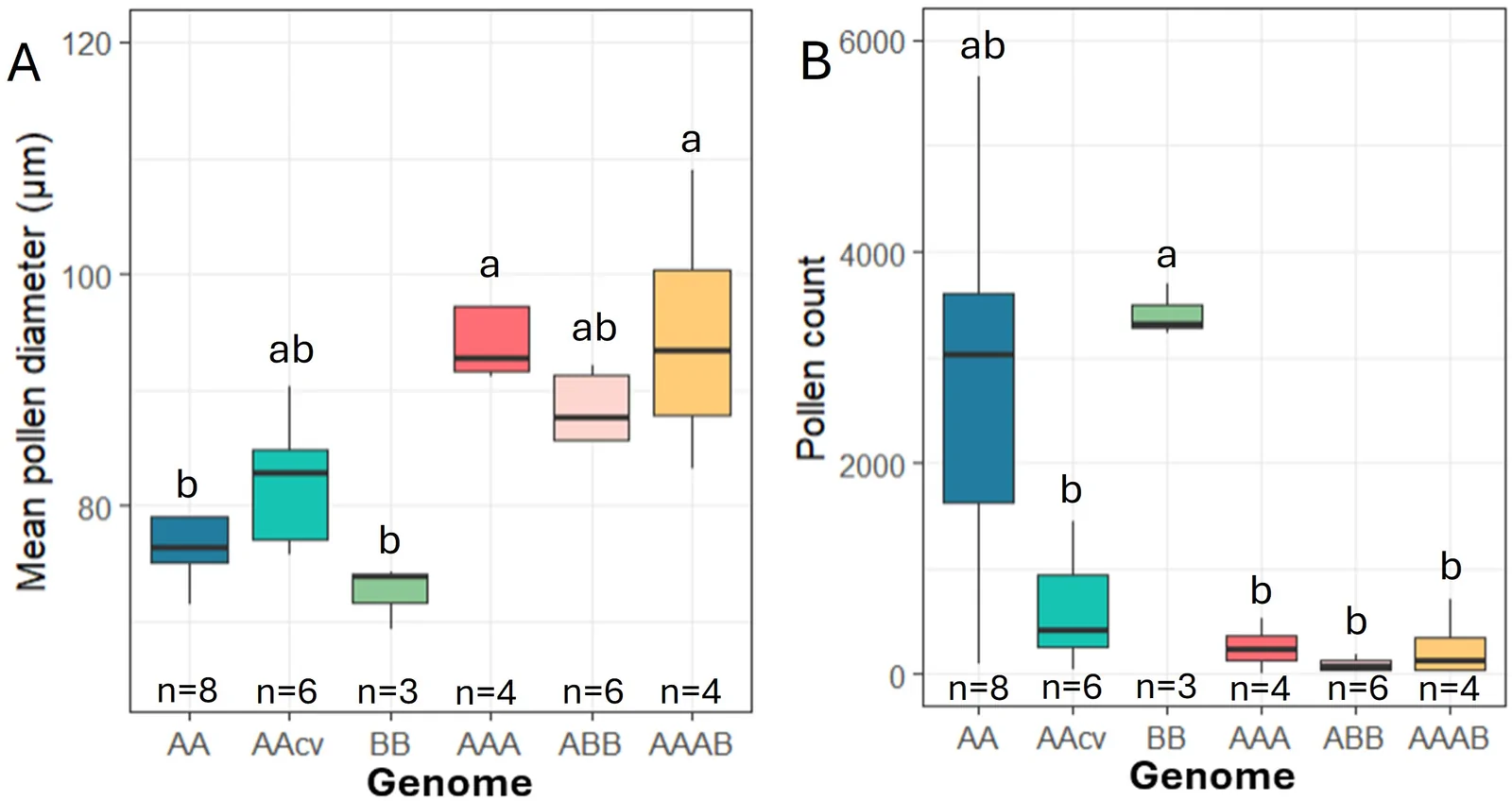
Distributions of **(A)** mean pollen diameter (µm) and **(B)** the amount of measured pollen grains for the AA, AAcv, BB, AAA, ABB and AAAB genomic groups sampled in Brazil. Significant differences according to *post-hoc* Wilcoxon tests are indicated by distinct letters in the graphs. The amount of genotypes assessed for a genome group is denoted below the bar corresponding to that genome.

### Variation in pollen diameter distributions within genome groups

Pollen diameter distributions of individual genotypes sampled from Tanzania and Brazil (**Supplementary Figures S6, S7** respectively), showed diverse distribution shapes. In plants, pollen diameter distributions provide information on the putative ploidy levels of the pollen produced by a certain genotype. Four main distribution types were identified among the different *Musa* genotypes via the multimode package in R. First is the narrow, strictly unimodal pollen diameter distribution in which pollen have very uniform sizes. Second is the unimodal distribution skewed towards smaller and/or larger pollen grain diameters, indicating that though the majority of pollen still falls within a certain size range, some deviating pollen grains are formed. Third is the bimodal distribution which indicates the presence of multiple pollen ploidy levels, and fourth is the broad multimodal distribution that indicates the formation of aneuploid pollen among euploid pollen. A representative selection of pollen diameter distributions for the different genome groups is displayed in **Figure 9**.

**Figure 9:**
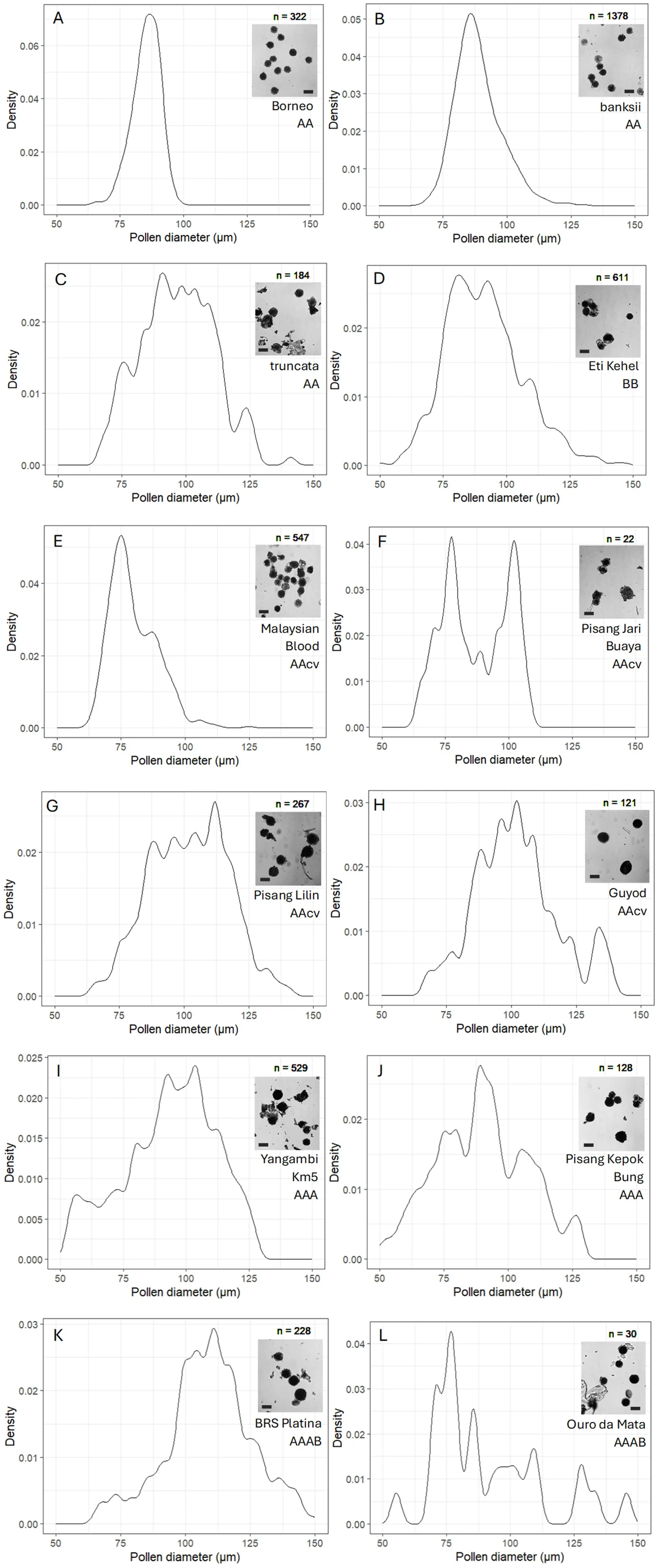
Representative pollen diameter density distributions for different *Musa* genotypes belonging to different genome groups, including **(A)** ‘Borneo’ (AA), **(B)** ‘*M. a.* ssp. banksii’ (AA), **(C)** ‘truncata’ (AA), **(D)** ‘Eti Kehel’ (BB), **(E)** ‘Malaysian Blood’ (AAcv), **(F)** ‘Pisang Jari Buaya’ (AAcv), **(G)** ‘Pisang Lilin’ (AAcv), **(H)** ‘Guyod’ (AAcv), **(I)** ‘Yangambi Km5’ (AAA), **(J)** ‘Pisang Kepok Bung’ (ABB), **(K)** ‘BRS Platina’ (AAAB) and **(L)** ‘Ouro Da Mata’ (AAAB). Pollen samples presented in **(A–H)** were collected in Tanzania, those presented in **(I–L)** were obtained from Brazil. Representative pictures of the rehydrated pollen grains for the different genotypes are shown together with their respective distribution, as well as the measured amount of pollen grains (n). Scale bars are 50 µm.

In general, wild diploid AA and BB genotypes exhibit a narrow unimodal distribution like ‘Borneo’ (**Figure 9A**), but could also display unimodal distributions with skewness at one or both ends such as ‘*M. a.* ssp. banksii’ (**Figure 9B**). A few wild genotypes displayed broad, multi-modal pollen diameter distributions, like AA ‘truncata’ (**Figure 9C**) and BB ‘Eti Kehel’ (**Figure 9D**). Similarly, pollen size distributions of many AAcv genotypes appeared unimodal or skewed unimodal (**Supplementary Figure S6**). For some genotypes a bimodal distribution was observed, such as for ‘Malaysian Blood’ (**Figure 9E**) and ‘Pisang Jari Buaya’ (**Figure 9F**). Many of the AAcv genotypes, however, had a broad, multimodal pollen diameter distribution as can be seen for ‘Pisang Lilin’ (**Figure 9G**) and ‘Guyod’ (**Figure 9H**). For the triploid AAA and ABB groups, all genotypes showed a broad multimodal pollen diameter distribution, as can be seen for AAA ‘Yangambi Km5'’ (**Figure 9I**) and ABB ‘Pisang Kepok Bung’ (**Figure 9J**) and the other triploid genotypes (**Supplementary Figure S7**). Similarly, for AAAB tetraploid genotypes, pollen diameter distributions were multi-modal and broad, as shown for ‘BRS Platina’ (**Figure 9K**), ‘Ouro Da Mata (**Figure 9L**) and other tetraploid genotypes (**Supplementary Figure S7**), instead of the expected larger pollen peak associated with diploid pollen formation. Overall, when comparing the genotypes that were common between Tanzania and Brazil, their pollen size distributions were similar across both locations for five out of the seven genotypes (**Supplementary Figure S8**).

### Relationship between pollen size and ploidy

To investigate the relationship between pollen grain size and their ploidy level, we measured pollen nuclear DNA contents and pollen sizes in a subset of genotypes from different genome groups that showed distinct pollen size distributions (**Figures 10A–J**). This comprises ‘Borneo’ and ‘truncata’ of the AA group, ‘Malaysian Blood’, ‘Pisang Jari Buaya’ and ‘Pisang Lilin’ of the AAcv group, ‘Eti Kehel’ (BB), ‘Yangambi Km5’ (AAA), ‘Benedetta’ (ABB), ‘Ambrosia’ (AAAA) and ‘Ouro Da Mata’ (AAAB). Due to lack of fresh pollen, visible generative nuclei area following PI staining was assessed instead of flow cytometry.

**Figure 10:**
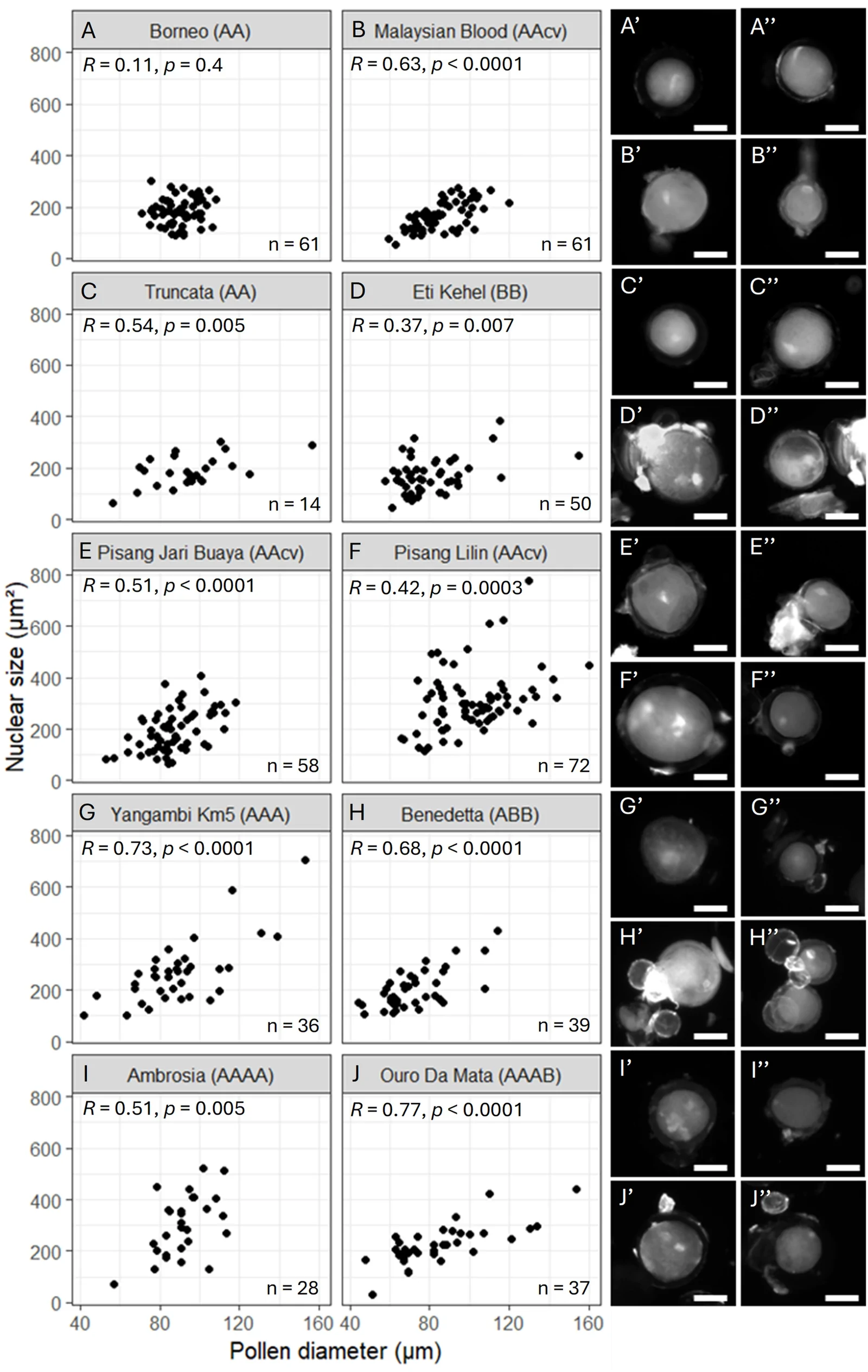
Relationship between pollen size and nuclear content in different *Musa* genotypes. **(A–J)** Scatter plots of pollen diameter (µm) in relation to the visible nuclear area (µm²) and **(A’–J’’)** representative images of PI-stained pollen grains for **(A, A’, A’’)** ‘Borneo’ (AA), **(B, B, B’’)** ‘Malaysian Blood’ (AAcv), **(C, C’, C’’)** ‘truncata’ (AA), **(D, D’, D’’)** ‘Eti Kehel’ **(BB)**, **(E, E’, E’’)** ‘Pisang Jari Buaya’ (AAcv), **(F, F’, F’’)** ‘Pisang Lilin’ (AAcv), **(G, G’, G’’)** ‘Yangambi Km5’ (AAA), **(H, H, H’’)** ‘Benedetta’ (ABB), **(I, I’, I’’)** ‘Ambrosia’ (AAAA), **(J, J’, J’’)** ‘Ouro Da Mata’ (AAAB). Scale bars; 50 µm.

A moderate but significant positive correlation between the visible generative nuclear area and the pollen diameter was found (*R* = 0.53, *p* < 0.0001) (**Supplementary Figure S9**). Also in the separate genotypes, significant positive correlations existed between pollen size and visible area, except for ‘Borneo’ (**Figure 10**). Fixated ‘Borneo’ displayed a pollen diameter distribution between 70-110 µm, and nuclear sizes between 100-300 µm² (**Figure 10A**). The three-fold difference in observed nuclear area within one ploidy level is postulated to be a consequence of size underestimation due to the elongated shape of *Musa* pollen generative nuclei (**Figure 10A’, A’’**). To estimate the maximum visible area of diploid pollen nuclei, the largest haploid nuclei size (300 µm²) was taken as a standard and by doubling the size of the sphere corresponding to this area, the maximum area of diploid nuclei would be 477 µm². Following the same approach triploid nuclear area would be 615 µm² and tetraploid pollen nuclei would be 759 µm² In order to estimate the percentages of higher ploidy pollen based on nuclear size, haploid pollen nuclei were estimated to be situated between 100-300 µm², diploid pollen nuclei between 301-477 µm³, triploid between 478-615 µm² and tetraploid between 616-759 µm². Only pollen with a nuclear area below 100 µm² was considered to be aneuploid in this approach.

In ‘Malaysian Blood’ the correlation between pollen diameter and pollen nuclear size was relatively strong (*R* = 0.63), with 11.5% (n = 61) of pollen with nuclei below 100 µm² having diameters around 60 µm, and 88.5% of pollen with haploid nuclei being situated between 65-120 µm diameter (**Supplementary Table S7**). However, in the diploids ‘truncata’, ‘Eti Kehel’, ‘Pisang Jari Buaya’ and ‘Pisang Lilin’, the correlations between pollen diameter and nuclear size were moderate (*R* = 0.54, *R* = 0.37, *R* = 0.51 and *R* = 0.42, respectively). Pollen with nuclei in the haploid size range in these genotypes also mainly fell within 70-120 µm diameter, but the higher ploidy pollen was not clearly related to a certain pollen diameter. For example, in ‘truncata’ (AA) and ‘Eti Kehel’, 7.1% (n = 14) and 6.0% (n = 50) of pollen had nuclear sizes above 300 µm², but these all had pollen diameters below 120 µm. The largest pollen grains in these genotypes (150-160 µm diameters) did not show nuclear sizes above 300 µm². Also, in ‘Pisang Jari Buaya’, pollen grain diameters did not exceed 120 µm, though 10.3% (n = 58) of pollen in this genotype had nuclear sizes within the diploid range. In ‘Pisang Lilin’, 33.3% (n = 72) of measured pollen nuclei fell into the diploid range (**Figure 10F**). Surprisingly it also showed 4.2% in the triploid range and 2.8% in the tetraploid range, indicating the occurrence of double meiotic restitution. Some diploid nuclei were found with pollen diameters of 80-90 µm, and others with 130-160 µm. In the triploids ‘Yangambi Km5’ (**Figure 10G**) and ‘Benedetta’ (**Figure 10H**), the correlation between nuclear size and pollen diameter was much stronger (*R* = 0.73 and *R* = 0.68, respectively). These triploids showed no pollen with a nuclear size less than 100 µm², though the only reason for this is that the abundant small, aborted pollen grains in these genotypes showed no nuclear staining and could therefore not be included in this assay. This being considered, most of the pollen grains that did show nuclei, fell into the haploid range, with 75.0% (n = 36) in ‘Yangambi Km5’ and 89.7% (n = 39) in ‘Benedetta’. They both also showed nuclei in the diploid range of 19.4% in ‘Yangambi Km5’ and 10.3% in ‘Benedetta’. ‘Yangambi Km5’ also showed 2.8% of pollen nuclei in the triploid range, indicating a low occurrence of meiotic restitution. As was already expected based on the broad pollen diameter distributions observed for the tetraploids under study, the tetraploids assessed here did not produce exclusively diploid pollen. Instead, ‘Ambrosia’ produced 50.0% (n = 28) pollen with nuclei in the diploid size range, 39.3% pollen with nuclei in the haploid range, and also low percentages of pollen with aneuploid (3.6%) and triploid (7.1%) nuclear sizes. ‘Ouro Da Mata’ showed only 8.1% (n = 37) pollen with nuclei in the diploid range, a majority of pollen with nuclei in the haploid range (86.5%) and a small percentage of pollen with nuclei in the aneuploid size range (5.4%). In ‘Ambrosia’ the correlation between pollen size and nuclear size was again moderate (*R* = 0.51), with all of its pollen with nuclei in the diploid size range having diameters between 80 and 120 µm. In ‘Ouro Da Mata’, the correlation between pollen size and nuclear size was stronger (*R* = 0.77) with most pollen nuclei in the diploid size range found in pollen with diameters above 110 µm.

Though the nuclear staining method used here has limitations, the data show that there is a general correlation between nuclear size and pollen diameter in *Musa* spp. Whereas no absolute relation between pollen diameter and the putative ploidy level of a pollen grain could be determined, in general, haploid pollen was found between 60-120 µm diameter.

### Prevalence of aneuploid and higher ploidy pollen in *Musa* spp.

To estimate the percentages of aborted aneuploid pollen and of higher ploidy pollen in an easy and high-throughput manner, we used pollen grain size as a proxy with a cut-off pollen diameter of 60 µm below which pollen grains were considered aneuploid and unviable, and a cut-off pollen diameter of 120 µm above which they were considered polyploid (diploid and higher).

The percentages of smaller and larger pollen detected for each genotype are given in **Supplementary Tables S8** and **S9**. The mean and standard deviation of the percentage of larger and smaller pollen production per genome group are shown in **Figures 11A, B**, respectively for Brazil samples and **Supplementary Figure S5** for Tanzania samples. A small significant difference in the percentage of larger pollen existed between the different genome groups sampled from Brazil (*χ²5* = 11.3, *p* = 0.046), but as there were major differences in larger pollen production between genotypes within a genome group, no statistically significant differences were observed between genome groups when performing Wilcoxon *post-hoc* tests. Larger pollen percentages were on average close to zero in AA and BB genome groups. AAcv genotypes had an average percentage of larger pollen of 2.84%. Tetraploid AAAB genotypes showed the highest average percentage of larger pollen with 10.09%, followed by AAA and ABB genotypes with 7.25% and 7.09% respectively (**Figure 11A**). The data obtained from Tanzania corroborated these results. No significant difference in average larger pollen grain production was observed between AA and AAcv groups (*χ²2* = 2.5, *p* = 0.09), though the average larger pollen formation in the AAcv group was higher than in the AA group (**Supplementary Figure S5C**). For five out of the seven common genotypes between Brazil and Tanzania, the larger pollen production percentage was highly similar. However, for AAcv genotypes ‘Pisang Kermian’ and ‘Pisang Lilin’, substantial differences in larger pollen production were observed between both locations. In Tanzania, ‘Pisang Lilin’ produced 11.6% larger pollen whereas it only produced 0.8% larger pollen in Brazil. In contrast, ‘Pisang Kermian’ produced 7.9% of larger pollen in Brazil, and none (0.0%) in Tanzania (**Supplementary Tables S8**, **S9**). Though this may partly be due to differences in pollen diameter between both locations, it indicates that the environmental conditions connected to a location, influence the production of higher ploidy pollen in these genotypes. No statistically significant differences were detected in the production of smaller pollen grains between genome groups in Brazil (*χ²5* = 5.4, *p* = 0.49) (**Figure 11B**) or in Tanzania (*χ²2* = 4.6, *p* = 0.10) (**Supplementary Figure S5D**).

**Figure 11:**
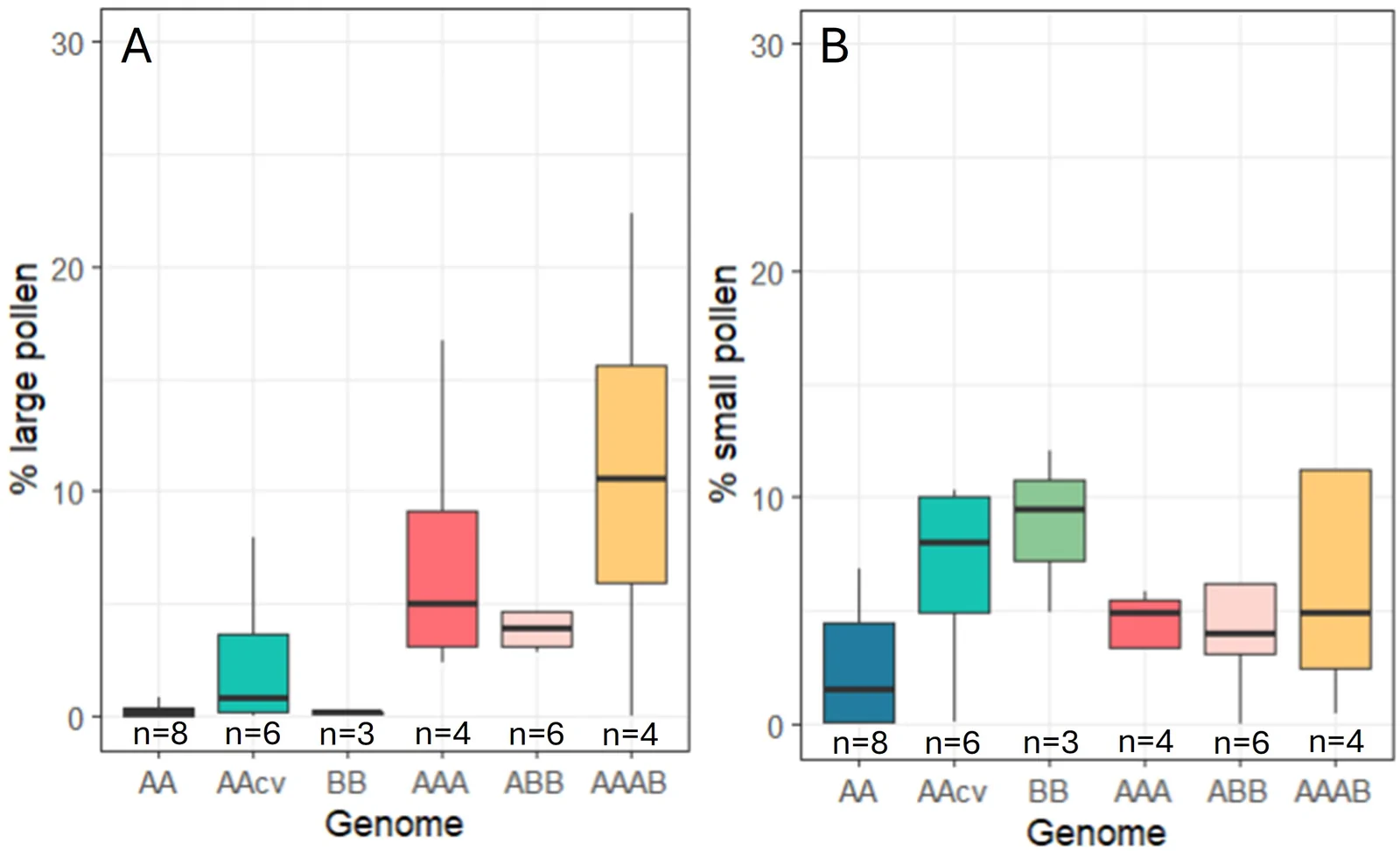
Percentages of **(A)** large pollen grains (diameter > 120 µm) and **(B)** small pollen grains (diameter > 120 µm) for **(A)** *Musa* genotypes belonging to AA and AAcv genomic groups sampled in Tanzania and **(B)** *Musa* genotypes of AA, AAcv, BB, AAA, ABB and AAAB genomic groups sampled in Brazil.

For the diploid genotypes of which the pollen nuclear content was determined, a significant correlation existed between the percentage of pollen nuclei larger than 300 µm² (higher ploidy pollen) and pollen diameters above 120 µm (*R* = 0.86, *p* = 0.026) (**Supplementary Figure S10**). For the triploid and tetraploid genotypes no significant correlation was found due to the confounding effects of their higher ploidy level. No significant correlation existed between the percentage of pollen nuclei smaller than 100 µm² and the percentage of pollen grains smaller than 60 µm in diploids, triploids and tetraploids (**Supplementary Figure S10**). This was expected as many of the small, aborted pollen grains did not show nuclear staining.

### Pollen size in relation to meiotic outcome and structural heterozygosity

Lastly, we investigated to what extent the amount of measured pollen and the percentage of pollen grains larger than 120 µm, or smaller than 60 µm, are determined by the outcome of meiosis, or by structural genetic features inherent to the genotypes, such as heterozygosity, the amount of different ancestor contributions and the amount of translocations. The genotypes and all data obtained from previous publications used in this analysis are listed in **Supplementary Table S4**.

We hypothesized the percentage of small pollen (<60 µm diameter) to be related to the levels of unbalanced tetrads and polyads found in these genotypes, as these indicate the formation of aneuploid pollen. Whereas no significant effect was found of the percentage of unbalanced tetrads, the percentage of polyad formation was positively associated with the percentage of small pollen among the wild AA group (*β* = 0.19, *SE* = 0.05, *p* = 0.02). Among the other genome groups, none of the meiotic end product types displayed significant effects on the percentage of small pollen. Next, the percentage of larger pollen (>120 µm diameter), a proxy for the presence of higher ploidy pollen, was tested for its correlation to the percentage of meiotic restitution products found in tetrad stage. Only for AAcv genotypes, the percentage of restituted products was positively associated with the percentage of larger pollen (*β* = 1.7, *SE* = 0.7, *p* = 0.04). For the other genome groups, none of the tetrad stage variables displayed a significant effect on the percentage of larger pollen. The amount of pollen measured per sampled inflorescence can be interpreted as an indication of the potential fertility of a genotype. The parameters included in the model were ploidy level, percentage of heterozygosity, amount of ancestries and the amount of translocations in a certain genotype. For the amount of pollen measured in AA and AAcv genotypes, only the amount of contributing ancestries showed a negative correlation(*β* = 12.8, *SE* = 3.7, *p* = 0.002). On the percentage of large pollen, only ploidy level had a positive significant effect (*β* = -346.2, *SE* = 156.1, *p* = 0.03). The amount of heterozygous translocations showed a positive significant effect on the percentage of small pollen (*β* = 1.6, *SE* = 0.7, *p* = 0.02) in AA and AAcv genotypes. It is important to mention that our linear regression model is inherently simplified. As genotypes from highly diverse groups were combined (i.e. AA, AAcv, AAA, AAAA), the nested variable such as ploidy and heterozygosity cannot be fully disentangled, meaning that our results reflect associations rather than strictly isolated causal effects.

## Discussion

The stability of male meiosis in different genotypes belonging to different genome groups was evaluated with the aim to gain insights into the underlying cellular defects/mechanisms through DAPI-stained chromosome spreading. ‘Borneo’ and ‘balbisiana’ were used as AA and BB reference due to their stable meiotic cell division. As reflected by the sharp unimodal pollen size distributions and the near 98% balanced tetrads these genotypes displayed, few meiotic aberrations were observed. These included an occasional laggard during anaphase II in ‘Borneo’ or during anaphase I in ‘balbisiana’. A cluster of three bivalents was seen in both BB genotypes in 30% of observed diakinesis meiocytes, whereas this was not observed in AA genotypes, indicating this is inherent to *M. balbisiana*. The presence of a heterozygous translocation can mediate the connection between non-homologous chromosome pairs due to recombination in the translocated region (Curtis et al., 2009; Miller, 2020). However, BB genotypes are homozygous for a 1/3 translocation which should result in normal homologous pairing (Anton et al., 2008; Baurens et al., 2018; Šimoníková et al., 2020). Moreover, as during subsequent chromosomal divisions no anaphase bridges were present in ‘balbisiana’, which is a hallmark of non-homologous recombination (Finardi et al., 2020), non-homologous recombination was likely not the cause for the observed bivalent clustering in BB genotypes.

Not all wild AA and BB genotypes had stable meiosis, and varying amounts of polyads were formed, i.e. 8.8% in ‘banksii’ (AA) and 51.8% in ‘Eti Kehel’ (BB). On the mature pollen level, this coincided with a skewed unimodal pollen size distribution for ‘banksii’, and a broad multimodal pollen size distribution for ‘Eti Kehel’, with 14.0% of aneuploid pollen nuclei. Both genotypes display higher frequencies of meiotic abnormalities as compared to the reference AA and BB lines, with in particular the presence of univalents in diakinesis and metaphase I as well as lagging bivalents in anaphase I. Additionally in ‘Eti Kehel’ chromosome bridges were observed during anaphase I that persisted up to telophase I, possibly explaining the increased severity of polyad formation over ‘banksii’ (Pampalona et al., 2016). Anaphase I bridges indicate for non-homologous recombination in ‘Eti kehel’. The karyotype of ‘Eti Kehel’ is unknown. Possibly ‘Eti kehel’ carries an additional heterozygous translocation on top of the homozygous 1/3 translocation in *M. balbisiana*, that could lead to non-homologous recombination. Structural heterozygosity is however most likely not the cause for the defects observed in ‘banksii’, as examined genotypes belonging to this subspecies have so far only shown the ancestral karyotype without translocations (Martin et al., 2023b). As only one to two univalents or lagging bivalents were found in the meiocytes of ‘banksii’ and ‘Eti Kehel’, it does seem unlikely that a genetic mutation underlies these defects, as they would affect more chromosome pairs in the latter case. Most likely, the meiotic defects recorded here were the consequence of mild environmental stress, as for example heat stress has been reported to cause univalents, non-homologous recombination and lagging bivalents in various other plant species (Loidl, 1989; Pécrix et al., 2011; De Storme et al., 2012; Higgins et al., 2012; De Storme and Geelen, 2020; Schindfessel et al., 2023).

More frequently than in AA genotypes, AAcv genotypes produced a low base level of polyads of 2.8% on average. Meiotic chromosome dynamics in ‘Pisang Jari Buaya’, displaying 7.6% polyad formation and 12.1% aneuploid pollen formation, were assessed to determine if different cellular defects occur as compared to wild AA genotypes. It was hereby noticeable that ‘Pisang Jari Buaya’ exhibited an increased diversity of meiotic aberrations, as well as a higher intensity of meiotic defects that were already visible in AA genotypes. Whereas in ‘banksii’ one to two lagging bivalents were observed in anaphase I, in ‘Pisang Jari Buaya’ up to seven lagging bivalents were found in anaphase I. Moreover, lagging chromosomes were also recorded in anaphase II, which was not seen in wild AA genotypes. This latter could indicate a recurring defect in adhesion of chromosomes to the spindle figure through the kinetochore complex (Li and Dawe, 2009; Zhou et al., 2023), or reduced motility of the spindle figure (Silkworth and Cimini, 2012). We hypothesize that in some meiocytes, where the majority of chromosomes remained around the equational plane, cytokinesis could not take place, resulting in occasional 2n pollen formation, as was previously described in grasshopper spermatocytes (Rebollo and Arana, 2001). The latter mechanism could also explain the low fraction of 2n pollen observed in the AAcv genotype ‘Pisang Jari Buaya’ (0.7% meiotic restitution, 10.3% diploid pollen). Another striking defect in ‘Pisang Jari Buaya’ is the occurrence of univalents in 28.1% of metaphase I meiocytes, indicating the absence of crossover formation between homologous chromosomes. This could be due to its complex hybrid genome configuration. 50% of its genome originates from *Musa halabanensis*, whereas the other half is mostly attributed to *M. acuminata* ssp. *banksii*, followed by *M. acuminata* ssp. *zebrina*, and a small contribution from *Musa schizocarpa* (Martin et al., 2023b). Intra- and interspecific hybrids typically show reduced homolog pairing and recombination because of sequence differences between the two parental genomes. The main assumption hereby is that the more the two parents are phylogenetically distant, the less homolog pairing and recombination will occur, leading to increased levels of univalents and associated induction of restitution (Braz et al., 2021; Forejt and Jansa, 2023).

Among the assessed AA and AAcv genotypes, ‘Pisang Lilin’ was the only genotype showing a substantial level of meiotic restitution. Also in previous literature, it has been reported as a consistent 2n pollen producer (Ortiz, 1997; Ssebuliba et al., 2008). This genotype could therefore have direct use in banana breeding programs, as it has desirable fruit characteristics, resistance against black sigatoka (Vuylsteke et al., 1993), and due to its 2n gamete production it could directly generate triploid progeny in crosses with other improved diploids. For the other genotypes analyzed, the low level of meiotic restitution seemed merely to be an artefact of occasional meiotic aberrations, such as lagging chromosomes as outlined above. Based on our preliminary observations, there are indications for the occurrence of premeiotic doubling in ‘Pisang Lilin’, as shown by the occurrence of 1.7% enlarged meiocytes in this genotype. No tetraploid meiocytes were observed among the meiotic chromosome spreads that could confirm this, but this could possibly be attributed to the limited amount of observations. Most of the meiotic defects observed in this genotype could be related to its genomic make-up and structural heterozygosity (Šimoníková et al., 2020; Martin et al., 2023b). Partial asynapsis was noticeable in 21.6% of the assessed pachytene meiocytes, coinciding with the hybrid nature of ‘Pisang Lilin’. Most of the ‘Pisang Lilin’ genome is attributed to *M. acuminata* ssp. *malaccensis*, with smaller contributions of *M. acuminata* ssp. *banksii*, *M. acuminata* ssp. *zebrina* and *Musa halabanensis* (Martin et al., 2023b). In parallel, in 56.5% of diakinesis-stage PMCs, clusters of three bivalents were observed, with often even two of these clusters in one single cell. The presence of a heterozygous 1/4 translocation (Šimoníková et al., 2020) could explain the clustering of chromosome one and four, but not the clustering of a third bivalent, and certainly not the occurrence of a second cluster of three trivalents, This could suggests the existence of additional undescribed translocations or other chromosome structural variations in ‘Pisang Lilin’, but as the high incidence of meiocytes showing bivalent clustering does not coincide with the 5% of anaphase bridges in MI, non-homologous recombination is probably not the main mechanism of bivalent clustering, but rather defective chromosome condensation or defective telomere bouquet or centromere clustering (Howard-Till and Loidl, 2018; Tian et al., 2020). Aside from 9% of metaphase I meiocytes that showed a few univalents and 25% of anaphase I cells where a few lagging chromosomes were observed, MI proceeded rather normally. Shepherd (1999) reported slightly higher incidences of meiotic defects during MI in ‘Pisang Lilin’, with 22.4% of meiocytes with chromosomal bridges during anaphase I and 26.5% of metaphase I meiocytes with univalents. This difference could be attributed to different environmental conditions, as also Shepherd (1999) noted differences in the prevalence of univalents in ‘Pisang Lilin’ depending on the location where samples were obtained. No previous reports on MII in ‘Pisang Lilin’ were found, however, in our samples MII seemed more disturbed than MI. During anaphase II, a substantial fraction of ‘Pisang Lilin’ meiocytes (23.1%) showed the presence of one to two lagging chromosomes, whereas another 38.5% exhibited one to two anaphase bridges, indicating failure of physical separation of sister chromatids. The presence of a few lagging chromosomes or anaphase bridges during MI and MII is however more indicative of polyad formation than of meiotic restitution (Bomblies et al., 2015; Shin et al., 2021; Silkova et al., 2021). Perhaps the complete omission of MI or MII is responsible as this is undetectable through chromosome spreading and leads to balanced dyads, as was mostly seen among the meiotic restitution products in ‘Pisang Lilin’.

The cytological evidence of ‘Pisang Lilin’ suggests a dual mechanism for 2n gamete formation. The observation of 1.7% enlarged meiocytes during tetrad stage points towards pre-meiotic genome doubling. At the same time, the presence of anaphase bridges and lagging chromosomes during both meiosis I and II indicates the occurrence of a meiotic restitution mechanism in parallel, hypothesized to be driven by failure of sister chromatid separation or a complete omission of one meiotic division. Supporting this is the observation of 16.7% higher ploidy pollen nuclei, including small amounts of triploid and even tetraploid pollen grains. This is more than expected based only on the 7.1% restituted products observed during tetrad stage, which would only result in 3.6% of diploid pollen in the pollen population. Occasional premeiotic doubling may increase the percentage of diploid pollen and a combination of premeiotic doubling and meiotic doubling may lead to the formation of tetraploid pollen. The presence of two different mechanisms of 2n gamete formation may also explain the unusual separation between diploid pollen with 80-100 µm diameters, and with diameters above 120 µm.

Parallel to diploid genotypes, we also investigated differences in meiotic stability among triploid genotypes. All triploids produced minimally 10% of polyads and 60-90% of balanced tetrads, indicating regular meiotic cell division with both MI and MII. This was unexpected as meiosis in triploids is inherently unbalanced due to uneven segregation of the three chromosome copies during MI (McClintock, 1928; Mather, 1935; Shepherd, 1999). Only ‘Williams Bell’ (AAA) produced almost exclusively polyads (88.7%). Nevertheless many small, aborted pollen grains were encountered in the broad multimodal pollen size distributions of all assessed triploids, indicating the presence of hidden aneuploidy in the balanced tetrads (Zhang et al., 2013).

Among the few well-formed pollen grains that displayed nuclei in ‘Yangambi Km5’ (AAA) and ‘Benedetta’ (ABB), most were in the haploid and diploid size range. In ‘Yangambi Km5 also few pollen with nuclei in the triploid size range were observed, coinciding with the small amount of restituted meiotic products visible in this genotype (1.3%). No pollen with nuclei in the triploid size range were encountered in ‘Benedetta’, as it also showed very little meiotic restitution products (0.4%). Most AAA and ABB genotypes showed small amounts of meiotic restitution, only ‘Enzirabahima’ and ‘Pisang Kepok Bung’ produced considerable amounts of restituted products (10.1% and 28.6% respectively). This indicates that 2n gamete formation is rather determined by mutations in specific genotypes, than related to genome group or ploidy level.

Male meiotic chromosome behavior in ‘Enzirabahima’ and ‘Williams Bell’ were studied more closely to discover the cytological basis for observed differences in meiotic restitution and polyad formation in the AAA triploid background. The high levels of polyads seen in ‘Williams Bell’ can be explained by the almost complete lack of pairing between homologous chromosomes, leading to a high number of univalents in MI, together with a lack of cohesion between sister chromatids in MII. Both ‘Cavendish’ and ‘Gros Michel’ subgroups contain highly heterozygous genomes with large contributions from *M. acuminata* ssp. *banksii*, *M. acuminata* ssp. *malaccensis*, *M. acuminata* ssp. *zebrina*, a so far unknown *M. acuminata* ssp., and minor contributions from *M. schizocarpa* and *M. halabanensis* (Martin et al., 2023b). It contains a lot of chromosomal structural variation, as it is heterozygous for three translocations; namely 1/4, 3/8, and 1/7 (Martin et al., 2023b). Meiosis in ‘Enzirabahima’ started with pairing between homologous chromosomes in the form of eleven trivalents. Presumably the pairing between the three homologues results in aberrant metaphase plate formations in metaphase I, where one, two, or all three homologues may be attached to the same pole (Paliulis et al., 2022). Lagging chromosomes observed during anaphase I indicate that indeed not all chromosomes were attached properly to the spindle figure. Additionally, the occurrence of chromosome bridges in anaphase I indicates the improper resolution of crossovers within the trivalent, or possibly non-homologous crossover between trivalents. As a consequence, also here no clear mechanism could be found that explains the occurrence of meiotic restitution, unless chromosome entanglement is so severe that it is unresolved in some cells, preventing the reductional division to take place (Rebollo and Arana, 2001). ‘Enzirabahima’ belongs to the subgroup of EAHBs, which have a large contribution from *M. acuminata* ssp. *zebrina*, *M. acuminata* ssp. *banksii*, and minor contributions from *M. schizocarpa* and *M. halabanensis* (Martin et al., 2023b). It is homozygous for translocation 3/8 of *M. acuminata* ssp. *zebrina* (Martin et al., 2023b). The increased genome complexity and structural heterozygosity in ‘Williams Bell’ could therefore be the reason for the high degree of observed asynapsis, whereas the relatively less complex genome of ‘Enzirabahima’ resulted in a relatively stable meiotic outcome.

Finally, meiotic stability among tetraploid genotypes was assessed. While tetraploids can theoretically display stable meiosis depending on their hybridity (Jelenkovic and Harrington, 1971; Braz et al., 2021; Forejt and Jansa, 2023), the examined tetraploids exhibited polyad formation similar to that of the triploids, which is likely inherited from their ancestors. This disturbance of meiosis is also noticeable in the mature pollen size distributions of the tetraploids under study. The discrepancy between the high frequency of balanced tetrads and the lower occurrence of diploid pollen nuclei suggests these balanced tetrads often conceal aneuploidy. This corroborates previous findings that hybrid tetraploids primarily yield diploid progeny when used as pollen donors (Oselebe et al., 2006).

We also evaluated whether structural genetic factors, such as ploidy, ancestral contributions, heterozygosity, and translocations, could explain variations in meiotic stability. Polyad formation was significantly associated with ploidy level. Notably, the prevalence of meiotic restitution was unrelated to any of these structural parameters, supporting the hypothesis that 2n gamete formation is primarily driven by specific mutations in key meiotic genes (Ortiz, 1997; Li and Dawe, 2009; France et al., 2021; Pochon et al., 2023), rather than broad structural heterozygosity. As expected, mature pollen size generally increased with ploidy (Darlington, 1937; Bretagnolle and Thompson, 1995). We are the first to experimentally confirm a positive correlation between generative nuclear area and pollen diameter in Musa, though this relationship is not absolute, as variations such as large pollen containing small nuclei were occasionally observed. By establishing a baseline from the stable diploid reference ‘Borneo’, we demonstrated that the 120 µm and 60 µm diameter thresholds can serve as high-throughput indicators for higher ploidy pollen and gametophytic aneuploidy, respectively. Specifically, in cultivated diploid (AAcv) genotypes, the frequency of large pollen (>120 µm) was directly associated with the occurrence of meiotic restitution, whereas in triploid and tetraploid genotypes, increased pollen size was driven primarily by parental ploidy level rather than specific meiotic outcomes. In contrast the presence of small, aborted pollen (<60 µm) was positively associated with polyad formation in wild AA genotypes, and with structural heterozygosity (specifically, the number of heterozygous translocations) in both AA and AAcv groups. Furthermore, a higher number of predicted ancestral contributions negatively impacted total pollen production in these diploids. Overall, while the direct cytological mechanisms by which complex ancestries and translocations disrupt meiosis remain intricate, our findings indicate that structural heterozygosity acts as a significant limiting factor on overall male fertility in banana.

## Conclusion

To facilitate the study of meiotic stability in banana, we determined whether pollen size could be related to ploidy in banana, and if so whether the presence of large pollen could be related to meiotic restitution and the presence of small pollen to aneuploidy. Among the different ploidy levels assessed, only few genotypes showed large amounts of meiotic restitution products, indicating that rather than structural genetic factors, specific mutations in key meiotic genes might be responsible for a large part of 2n pollen production in banana genotypes. Among all diploid genotypes assessed, only ‘Pisang Lilin’ was found to consistently produce 2n pollen, possibly both through premeiotic doubling and meiotic restitution.

In general, larger nuclear sizes were associated with larger pollen grains. Moreover, the presence of pollen with a diameter above 120 µm was related to meiotic restitution in cultivated diploid genotypes, and the presence of pollen with a diameter below 60 µm was related to polyad formation in wild diploid genotypes.

## Data Availability

The original contributions presented in the study are publicly available. This data can be found here: https://rdr.kuleuven.be/dataset.xhtml?persistentId=doi:10.48804/T8JRT3.
