# Supplementary material for: Structural defects in male meiotic cell division lead to reproductive genome instability and disturbed microsporogenesis in banana (Musa spp.)
Source: Front Plant Sci. 2026 Sep 30;17:1923014. doi: 10.3389/fpls.2026.1923014 (PMC13662919; doi:10.3389/fpls.2026.1923014)
Supplement: Supplementary file 1 [file DataSheet1.docx]

**Table S1:** List of 71 genotypes sampled in Arusha, Tanzania, in September 2021, including the amount of inflorescences sampled for the genotypes (n). PNG denotes Papua New Guinea, n.a. denotes missing data.

| ITC code | Name | Origin | Type | Species  /group | Subspecies  /subgroup | n |
| --- | --- | --- | --- | --- | --- | --- |
| ITC0530 | A3617/9 | Fiji | wild | AA | n.a. | 3 |
| ITC0712 | AAcv Rose | Indonesia | edible | AAcv | n.a. | 3 |
| ITC1028 | Agutay | Philippines | wild | AA | n.a. | 1 |
| ITC0281 | Akondro Mainty | Madagascar | edible | AAcv | n.a. | 3 |
| ITC0884 | Awondaeke | PNG | edible | AAcv | n.a. | 2 |
| ITC0620 | Banksii | PNG | wild | AA | banksii | 1 |
| ITC1181 | Binaktong | Philippines | edible | AAcv | n.a. | 1 |
| ITC0253 | Borneo | Borneo | wild | AA | microcarpa | 3 |
| ITC0249 | Calcutta 4 | Myanmar | wild | AA | burmannicoides | 3 |
| ITC1193 | Diploide EMBRAPA205 | n.a. | seeded | AA | n.a. | 3 |
| ITC0271 | Eti Kehel | unknown | wild | BB | n.a. | 2 |
| ITC0798 | Garunga | PNG | edible | AAcv | n.a. | 1 |
| ITC0299 | Guyod | Philippines | edible | AAcv | n.a. | 2 |
| ITC0847 | Hova | PNG | edible | AAcv | n.a. | 1 |
| ITC0382 | Hybrid | unknown | wild | AA | n.a. | 3 |
| ITC1460 | Ijihu Inkundu | Tanzania | edible | AAcv | Mchare | 2 |
| ITC1468 | Kahuti | Tanzania | edible | AAcv | Mchare | 1 |
| ITC0788 | Katual Vunalir | PNG | edible | AAcv | n.a. | 3 |
| ITC0660 | Khae (Phrae) | Thailand | wild | AA | siamea | 1 |
| ITC0533 | Kluai Lep Mu Nang | Thailand | edible | AAcv | n.a. | 3 |
| ITC0840 | Kuspaka | PNG | edible | AAcv | n.a. | 2 |
| ITC0943 | Kwaro | PNG | edible | AAcv | n.a. | 1 |
| ITC0283 | Long Tavoy | unknown | wild | AA | burmannica | 3 |
| ITC0254 | Madang | unknown | wild | AA | malaccensis | 1 |
| ITC0728 | Maia Oa | unknown | wild | AA | zebrina | 1 |
| ITC1454 | Makyughu I | Tanzania | edible | AAcv | n.a. | 3 |
| ITC1446 | Makyughu II | Tanzania | edible | AAcv | Mchare | 3 |
| ITC0869 | Mala | PNG | edible | AAcv | n.a. | 3 |
| ITC0568 | Malaysian Blood | Malaysia | edible | AAcv | n.a. | 3 |
| ITC0809 | Maleb | PNG | edible | AAcv | n.a. | 2 |
| ITC0612 | Mambee Thu | PNG | edible | AAcv | n.a. | 1 |
| ITC1223 | Mshale | Tanzania | edible | AAcv | n.a. | 3 |
| ITC1455 | Mshale Mlelembo | Tanzania | edible | AAcv | Mchare | 1 |
| ITC0896 | Musa acuminata banksii | PNG | wild | AA | banksii | 3 |
| ITC1358 | Ngu | Vietnam | edible | AAcv | Sucrier | 1 |
| ITC0717 | Nzumoheli I | Comoros | edible | AAcv | n.a. | 3 |
| ITC0668 | Pa (Musore) no.2 | Thailand | wild | AA | n.a. | 3 |
| ITC0406 | Pa Musore no.3 | Thailand | wild | AA | n.a. | 1 |
| ITC0408 | Pa Songkhla | Thailand | wild | AA | n.a. | 3 |
| ITC0609 | Pahang | Malaysia | wild | AA | malaccensis | 3 |
| ITC1254 | Paka | Zanzibar | edible | AAcv | n.a. | 3 |
| ITC1229 | Pamoti On | Philippines | edible | AAcv | n.a. | 3 |
| ITC0611 | Pisang Berlin | Indonesia | edible | AAcv | n.a. | 3 |
| ITC0480 | Pisang Buntal | Malaysia (Borneo) | edible | AAcv | n.a. | 3 |
| ITC0415 | Pisang Cici Alas | Indonesia | wild | AA | n.a. | 3 |
| ITC0312 | Pisang Jari Buaya | Malaysia | edible | AAcv | n.a. | 2 |
| ITC0437 | Pisang Kermian | Malaysia | edible | AAcv | n.a. | 3 |
| ITC1345 | Pisang Kra | Malaysia | wild | AA | malaccensis | 3 |
| ITC1121 | Pisang Lilin | unknown | edible | AAcv | n.a. | 3 |
| ITC0258 | Pisang Madu | Malaysia | edible | AAcv | n.a. | 3 |
| ITC0685 | Pisang Pipit | Indonesia | edible | AAcv | n.a. | 3 |
| ITC0679 | Pisang Sapon | Indonesia | edible | AAcv | n.a. | 3 |
| ITC1349 | Pisang Serun 400 | Malaysia | wild | AA | malaccensis | 3 |
| ITC1348 | Pisang Serun 404 | Malaysia | wild | AA | malaccensis | 3 |
| ITC0294 | Pitu | PNG | edible | AAcv | n.a. | 3 |
| ITC0447 | Pu-te Wey | Malaysia (Borneo) | edible | AAcv | n.a. | 3 |
| ITC0434 | Racadag | Philippines | edible | AAcv | n.a. | 1 |
| ITC0629 | Selangor 2 | Thailand | wild | AA | n.a. | 3 |
| ITC1060 | Selangor | unknown | wild | AA | malaccensis | 3 |
| ITC1013 | Sena | PNG | edible | AAcv | n.a. | 3 |
| ITC0810 | Sihir | PNG | edible | AAcv | n.a. | 1 |
| ITC0266 | Sowmuk | PNG | edible | AAcv | n.a. | 2 |
| ITC0894 | Tainga | PNG | edible | AAcv | n.a. | 1 |
| ITC1004 | To'o | PNG | edible | AAcv | n.a. | 1 |
| ITC0393 | Truncata | Malaysia | wild | AA | truncata | 3 |
| ITC1231 | Tudlo Tumbaga | Philippines | edible | AAcv | n.a. | 2 |
| ITC0069 | type 2 | unknown | wild | AA | n.a. | 2 |
| ITC0373 | Uwati | PNG | edible | AAcv | n.a. | 1 |
| ITC0600 | Waimara | PNG | edible | AAcv | n.a. | 1 |
| ITC0774 | Yenai | PNG | edible | AAcv | n.a. | 2 |
| ITC0966 | Zebrina (G.F.) | unknown | wild | AA | n.a. | 3 |

**Table S2:** List of 31 genotypes sampled in Bahia, Brazil, in March 2022, including the amount of inflorescences sampled for the genotypes (n). No ITC codes were provided for most of the sampled genotypes. PNG denotes Papua New Guinea, n.a. denotes missing data.

| ITC code | Name | Origin | Type | Species /Group | Subspecies /subgroup | n |
| --- | --- | --- | --- | --- | --- | --- |
| n.a. | Malbut | unknown | wild | AA | n.a. | 3 |
| n.a. | Calcutta | Myanmar | wild | AA | burmannicoides | 3 |
| n.a. | PA Rayong | Thailand | wild | AA | siamea | 3 |
| n.a. | Pisang Lidi | unknown | edible | AAcv | n.a. | 3 |
| n.a. | Malaccensis (cova01) | Malaysia | wild | AA | malaccensis | 3 |
| n.a. | PA Musore 2 | Thailand | wild | AA | malaccensis | 3 |
| n.a. | Krasan Saichon | unknown | n.a. | AA | n.a. | 3 |
| n.a. | Selangor | unknown | wild | AA | malaccensis | 3 |
| n.a. | PA Patthalung | Thailand | edible | AAcv | n.a. | 3 |
| n.a. | Pisang Serum | Malaysia | wild | AA | malaccensis | 3 |
| n.a. | IDO 110 | unknown | edible | AAcv | n.a. | 3 |
| n.a. | Pisang Kermain | Malaysia | edible | AAcv | n.a. | 3 |
| ITC1121 | Pisang lilin | unknown | edible | AAcv | n.a. | 3 |
| n.a. | Uwati | PNG | edible | AAcv | n.a. | 3 |
| n.a. | BB França | unknown | wild | BB | n.a. | 3 |
| n.a. | Butuhan | Philippines | wild | BB | n.a. | 3 |
| n.a. | Balbisiana Franca | unknown | wild | BB | n.a. | 3 |
| n.a. | Grande Naine | unknown | edible | AAA | Cavendish | 3 |
| n.a. | Yangambi Km5 | unknown | edible | AAA | Ibota | 3 |
| n.a. | Khai Tong Ruong | Thailand | edible | AAA | Ibota | 3 |
| ITC0662 | Khai Thong Ruang | Thailand | edible | AAA | Ibota | 3 |
| n.a. | Yangambi N°2 | unknown | edible | AAB | Silk | 3 |
| n.a. | Moenang | Thailand | edible | ABB | n.a. | 3 |
| n.a. | Pisang Kepok Bung | Indonesia | edible | ABB | Peyan | 3 |
| n.a. | Benedetta | unknown | edible | ABB | Saba | 3 |
| n.a. | Saba Honduras | Honduras | edible | ABB | Saba | 3 |
| ITC0361 | Blue Java | unknown | edible | ABB | Ney Mannan | 3 |
| n.a. | BRS Platina | Brazil | edible | AAAB | n.a. | 3 |
| n.a. | BRS Princesa | unknown | edible | AAAB | n.a. | 3 |
| n.a. | Ouro da Mata | Brazil | edible | AAAB | n.a. | 3 |
| n.a. | FHIA 18 | Honduras | edible | AAAB | n.a. | 3 |

**Table S3:** List of 31 genotypes for which in total 71 male buds were collected and fixated, at varying locations and at varying dates in Bahia in March 2022. Each line represents a separate male bud. The developmental age of the male bud is given as the amount of scars on the rachis. The location of the tetrad stage is represented as ‘S’ followed by the number of flower clusters prior to the flower cluster in anthesis (S0). n.a. denotes missing data.

| Species  /Group | Subspecies /Subgroup | Name | Location | Date of fixation | Age male bud | Tetrad stage |
| --- | --- | --- | --- | --- | --- | --- |
| AA | malaccensis | malaccensis | Belgium | 11-09-2020 | n.a. | S28 |
|  |  |  |  | 16-10-2020 | n.a. | S27 |
|  |  |  |  | 12-04-2021 | 339 | S28 |
|  |  | Pa Musore | Tanzania | 9-06-2022 | 15 | S28 |
|  |  |  |  | 9-06-2022 | 51 | S27 |
|  |  | Pahang | Tanzania | 30-9-2021 | 24 | S33 |
|  | burmannicoides | Calcutta 4 | Belgium | 9-04-2021 | 171 | S30 |
|  |  |  | Uganda | 16-03-21 | 25 | S35 |
|  |  |  |  | 16-03-21 | 125 | S35 |
|  |  |  |  | 16-03-21 | 211 | S31 |
|  |  |  | Tanzania | 29-9-2021 | 135 | S38 |
|  |  |  |  | 29-9-2021 | 192 | S32 |
|  |  |  |  | 29-9-2021 | 147 | S36 |
|  | microcarpa | Borneo | Tanzania | 30-9-2021 | 180 | S30 |
|  |  |  |  | 30-9-2021 | 42 | S28 |
|  |  |  |  | 30-9-2021 | 24 | S31 |
|  |  |  |  | 14-06-2023 | 21 | S24 |
|  | zebrina | Maia Oa | Tanzania | 8-06-2022 | 45 | S27 |
|  |  |  |  | 8-06-2022 | 21 | S33 |
|  |  |  |  | 8-06-2022 | 78 | S27 |
|  | truncata | truncata | Tanzania | 9-06-2022 | 15 | S41 |
|  |  |  |  | 9-06-2022 | 51 | S41 |
|  |  |  |  | 15-06-2023 | 123 | S35 |
|  |  |  |  | 15-06-2023 | 45 | S38 |
|  | banksii | banksii | Tanzania | 8-06-2022 | 51 | S20 |
|  |  |  |  | 14-06-2023 | 95 | S22 |
| AAcv | n.a. | Malaysian Blood | Tanzania | 8-06-2022 | 51 | S25 |
|  |  |  |  | 8-06-2022 | 27 | S32 |
|  |  |  |  | 8-06-2022 | 15 | S30 |
|  | n.a. | Sena | Tanzania | 9-06-2022 | 84 | S29 |
|  |  |  |  | 9-06-2022 | 123 | S25 |
|  | n.a. | Morong Princesa | Tanzania | 9-06-2022 | 57 | S23 |
|  | n.a. | Guyod | Tanzania | 30-09-2021 | 48 | S32 |
|  | n.a. | Cv Rose | Tanzania | 30-9-2021 | 51 | S27 |
|  |  |  |  | 30-9-2021 | 33 | S25 |
|  |  |  |  | 30-9-2021 | 30 | S25 |
|  | n.a. | Pitu | Tanzania | 30-9-2021 | 72 | S24 |
|  |  |  |  | 30-9-2021 | 9 | S32 |
|  |  |  |  | 30-9-2021 | 54 | S25 |
|  | n.a. | Pisang Jari Buaya | Tanzania | 30-9-2021 | 117 | S36 |
|  |  |  |  | 1-10-2021 | 144 | S36 |
|  |  |  |  | 9-06-2022 | 129 | S35 |
|  | n.a. | Pisang Lilin | Tanzania | 29-9-2021 | 57 | S20 |
|  |  |  |  | 29-9-2021 | 75 | S25 |
|  |  |  |  | 29-9-2021 | 84 | S19 |
|  |  |  |  | 9-06-2022 | 36 | S29 |
|  |  |  |  | 14-06-2023 | 0 | S30 |
|  |  |  |  | 15-06-2023 | 99 | S26 |
|  | Mchare | Huti White | Tanzania | 1-10-2021 | 87 | S32 |
|  |  |  |  | 4-10-2021 | 27 | S33 |
|  |  |  |  | 4-10-2021 | 12 | S32 |
|  |  | Akondro Mainty | Tanzania | 1-10-2021 | 27 | S35 |
|  |  |  |  | 1-10-2021 | 36 | S35 |
|  |  |  |  | 1-10-2021 | 63 | S30 |
|  |  | Ijihu Inkundu | Tanzania | 1-10-2021 | 102 | S32 |
|  |  |  |  | 1-10-2021 | 108 | S33 |
|  |  | Mchare Mlelembo | Tanzania | 1-10-2021 | 54 | S34 |
|  |  |  |  | 1-10-2021 | 60 | S29 |
|  |  |  |  | 1-10-2021 | 93 | S25 |
|  |  | Mchare laini | Tanzania | 1-10-2021 | 9 | S38 |
| BB |  | balbisiana | Belgium | 17-03-2021 | 23 | S29 |
|  |  | Eti Kehel | Tanzania | 9-06-2022 | 75 | S30 |
| AAA | Cavendish | Williams Bell | Belgium | 11-09-2020 | n.a. | S33 |
|  | Ibota | Yangambi Km5 | Brazil | 22-03-2022 | 2 | S21 |
|  | Mutika/Lujugira | Enzirabahima | Uganda | 16-03-21 | 14 | S26 |
|  |  |  |  | 16-03-21 | 29 | S28 |
|  |  |  |  | 16-03-21 | 43 | S28 |
| ABB | Peyan | Pisang Kepok Bung | Brazil | 22-03-2022 | 37 | S21 |
|  | Saba | Benedetta | Brazil | 22-03-2022 | 65 | S19 |
| AAAA | n.a. | Ambrosia | Brazil | 22-03-2022 | 14 | S33 |
|  | n.a. | 917K-2 | Uganda | 16-03-21 | 16 | S28 |
|  |  |  |  | 16-03-21 | 33 | S29 |
|  |  |  |  | 16-03-21 | 50 | S30 |
| AAAB | n.a. | Ouro da mata | Brazil | 22-03-2022 | n.a. | S25 |

**Table S4:** Parameters of male meiotic stability and genetic structure for a subset of genotypes. Measured in this study were the standard deviation (SD) in pollen diameter, the percentage of larger pollen (diameter > 120 µm), the amount of measured pollen per sampled inflorescence (n pollen), the percentage of restituted products, the percentage of polyads and the percentage of unbalanced (UB) tetrads. For genotypes for which pollen data was available from both Tanzania and Brazil, the averages of pollen diameter standard deviation, percentage of larger pollen and the amount of pollen between both locations were used. The amount of ancestors and percentage heterozygosity were obtained from Martin et al. (2023), the amount of heterozygous translocations from Martin et al. (2020).

| Species  /Group | Name | Small pollen (%) | Large pollen (%) | n pollen | Restit.  (%) | Polyad  (%) | UB tetrads (%) | n ancestors | Hetero-zygosity (%) | N trans-locations |
| --- | --- | --- | --- | --- | --- | --- | --- | --- | --- | --- |
| AA | Borneo | 0.00 | 0.00 | 322 | 0.10 | 0.85 | 0.49 | 2 | 2.22 | 0 |
| AA | Pa Songkhla | 0.00 | 0.00 | 3222 | n.a. | n.a. | n.a. | 1 | 2.15 | 0 |
| AA | Calcutta 4 | 16.50 | 0.00 | 3441 | 1.75 | 1.55 | 3.26 | 1 | 2.39 | 0 |
| AA | Pisang Serun 400 | 0.04 | 0.00 | 2426 | n.a. | n.a. | n.a. | 4 | 3.07 | 1 |
| AA | Selangor | 3.42 | 0.15 | 1448 | n.a. | n.a. | n.a. | 1 | 1.87 | 0 |
| AA | Pisang Kra | 0.11 | 0.00 | 951 | n.a. | n.a. | n.a. | 3 | 4.07 | 0 |
| AA | Pisang Cici Alas | 0.00 | 0.05 | 2043 | n.a. | n.a. | n.a. | 1 | 1.24 | 0 |
| AA | Long Tavoy | 0.00 | 3.71 | 1862 | n.a. | n.a. | n.a. | 1 | 2.00 | 0 |
| AA | Zebrina (G.F.) | 0.00 | 0.00 | 220 | n.a. | n.a. | n.a. | 1 | 1.00 | n.a. |
| AA | Pahang | 0.00 | 0.77 | 261 | 0.10 | 0.34 | 0.10 | 1 | 1.40 | 0 |
| AA | Pisang Serun 404 | 7.63 | 0.46 | 245 | n.a. | n.a. | n.a. | 1 | 2.54 | 0 |
| AA | M. a. banksii | 0.00 | 0.73 | 1378 | n.a. | n.a. | n.a. | 1 | 0.30 | 0 |
| AA | Khae (Phrae) | 0.00 | 5.23 | 153 | n.a. | n.a. | n.a. | 1 | 0.65 | 0 |
| AA | Banksii | 2.04 | 0.00 | 48 | 0.06 | 8.84 | 0.28 | 1 | 0.27 | 0 |
| AA | Truncata | 0.00 | 5.98 | 184 | 1.16 | 7.20 | 1.12 | 3 | 1.93 | 0 |
| AA | Maia Oa | 0.34 | 14.83 | 290 | 0.11 | 0.91 | 0.93 | 1 | 1.01 | 0 |
| AA | Malaccensis | 0.08 | 0.83 | 3638 | 2.44 | 1.44 | 7.81 | 1 | 2.87 | n.a. |
| AA | Pa Musore | 0.03 | 3.01 | 1758 | 0.00 | 0.34 | 0.06 | n.a. | n.a. | n.a. |
| AA | Pa Rayong | 2.04 | 0.00 | 3576 | n.a. | n.a. | n.a. | 1 | 1.70 | 0 |
| AAcv | Racadag | 12.5 | 0.00 | 15 | n.a. | n.a. | n.a. | 5 | 4.35 | 2 |
| AAcv | Hova | 0.00 | 0.00 | 853 | n.a. | n.a. | n.a. | 6 | 4.18 | n.a. |
| AAcv | Pisang Sapon | 0.00 | 0.00 | 1064 | n.a. | n.a. | n.a. | 4 | 3.91 | 1 |
| AAcv | Sena | 0.00 | 0.07 | 1527 | 0.00 | 0.18 | 0.11 | 3 | 1.29 | 0 |
| AAcv | Katual Vunalir | 0.00 | 0.00 | 166 | n.a. | n.a. | n.a. | 4 | 2.70 | 2 |
| AAcv | Pisang Berlin | 0.31 | 0.00 | 954 | n.a. | n.a. | n.a. | 4 | 4.28 | 3 |
| AAcv | Paka | 0.00 | 0.50 | 202 | n.a. | n.a. | n.a. | 4 | 3.73 | 1 |
| AAcv | Malaysian Blood | 0.00 | 0.18 | 547 | 0.08 | 1.53 | 1.87 | 5 | 3.87 | 2 |
| AAcv | Pisang Madu | 0.00 | 0.41 | 246 | n.a. | n.a. | n.a. | 5 | 5.40 | 1 |
| AAcv | Pisang Pipit | 0.00 | 2.56 | 430 | n.a. | n.a. | n.a. | 5 | 4.26 | 2 |
| AAcv | Akondro Mainty | 0.00 | 3.80 | 158 | 1.27 | 5.54 | 1.59 | 5 | 4.23 | 2 |
| AAcv | Sowmuk | 3.80 | 0.64 | 157 | n.a. | n.a. | n.a. | 6 | 3.83 | 2 |
| AAcv | Guyod | 1.64 | 13.22 | 121 | 1.70 | 0.70 | 0.10 | 3 | 2.66 | 1 |
| AAcv | Pisang Buntal | 0.00 | 0.00 | 29 | n.a. | n.a. | n.a. | 6 | 3.89 | 1 |
| AAcv | Sihir | 0.00 | 0.80 | 376 | n.a. | n.a. | n.a. | 4 | 1.69 | 0 |
| AAcv | Pisang Jari Buaya | 0.00 | 0.00 | 22 | 0.72 | 7.60 | 0.05 | 4 | 5.49 | 0 |
| AAcv | Pisang Lilin | 5.18 | 6.21 | 259 | 7.11 | 4.57 | 1.07 | 4 | 4.06 | 1 |
| AAcv | CV Rose | 0.00 | 0.08 | 1326 | 0.18 | 0.96 | 0.47 | n.a. | n.a. | n.a. |
| AAcv | IjihuInkundu | 0.25 | 0.25 | 394 | 0.46 | 6.14 | 0.81 | n.a. | n.a. | n.a. |
| AAcv | Mchare Mlelembo | 0.00 | 4.41 | 68 | 0.86 | 5.25 | 1.05 | n.a. | n.a. | n.a. |
| AAcv | Pitu | 0.00 | 0.36 | 555 | 1.47 | 0.59 | 0.00 | n.a. | n.a. | n.a. |
| AAcv | Pa Patthalung | 9.09 | 4.55 | 44 | n.a. | n.a. | n.a. | 4 | 4.19 | 3 |
| BB | Balbisiana | 4.97 | 0.15 | 3323 | 0.63 | 1.51 | 0.18 | 1 | 1.79 | 0 |
| BB | Eti Kehel | 0.98 | 4.09 | 611 | 0.68 | 51.90 | 0.00 | 1 | n.a. | 0 |
| BB | Butuhan | 12.05 | 0.03 | 3686 | n.a. | n.a. | n.a. | 1 | 1.58 | 0 |
| AAA | Williams Bell | n.a. | n.a. | n.a. | 1.30 | 88.66 | 6.39 | 6 | 6.22 | 3 |
| AAA | Yangambi Km5 | 5.86 | 6.62 | 529 | 1.33 | 10.15 | 0.33 | 5 | 5.98 | 2 |
| ABB | Benedetta | 0.00 | 3.89 | 180 | 0.46 | 10.25 | 0.10 | n.a. | n.a. | n.a. |
| ABB | Pisang Kepok Bung | 6.25 | 4.69 | 128 | 28.63 | 27.33 | 11.37 | 4 | n.a. | n.a. |
| ABB | Saba Honduras | 4.00 | 28.00 | 25 | n.a. | n.a. | n.a. | 4 | 9.90 | 0 |
| AAAB | Ouro Da Mata | 6.67 | 13.33 | 30 | 0.71 | 15.65 | 0.00 | n.a. | n.a. | n.a. |

**Figure S1:** Pearson correlation tests between the presence of meiotic products over analyzed genotypes. (A) Correlation between percentages of dyads and polyads, (B) correlation between percentages of dyads and tetrads, and (C) correlation between percentages of polyads and tetrads.

**Figure S2:** Tetrad stage meiocytes in ‘Pisang Lilin’, larger sized meiocytes (72.76±1.03 µm compared to 50.50±6.35µm) are encircled, dyads are indicated with arrowheads, a polyad is indicated with an arrow.

**Table S5:** Quantification of meiotic defects in ‘Borneo’, *M. acuminata* ssp. *banksii*, *M. balbisiana* and ‘Eti Kehel’. For each variety, the aberration type as well as its relative abundance (%) is provided, together with the total amount of meiocytes observed in a certain meiotic stage (n).

| Meiotic stage | Defect type | AA | | | | BB | | | |
| --- | --- | --- | --- | --- | --- | --- | --- | --- | --- |
|  |  | Borneo | | banksii | | balbisiana | | Eti Kehel | |
|  |  | % | n | % | n | % | n | % | n |
| pachytene | Normal | 100.0 | 38 | 100.0 | 32 | 100.0 | 20 | 100.0 | 58 |
| diakinesis | Normal | 100.0 | 18 | 71.4 | 7 | 72.7 | 11 | 69.2 | 13 |
|  | Univalents | 0.0 |  | 14.3 |  | 0.0 |  | 0.0 |  |
|  | Trivalents | 0.0 |  | 14.3 |  | 0.0 |  | 0.0 |  |
|  | Clusters | 0.0 |  | 0.0 |  | 27.3 |  | 30.8 |  |
| metaphase I | Normal | 100.0 | 12 | 83.3 | 6 | 100.0 | 5 | 86.3 | 29 |
|  | Univalents | 0.0 |  | 16.7 |  | 0.0 |  | 10.3 |  |
|  | Nonhomologous recombination | 0.0 |  | 0.0 |  | 0.0 |  | 3.4 |  |
| anaphase I | Normal | 100.0 | 3 | 40.0 | 5 | 75.0 | 4 | 18.2 | 11 |
|  | Laggards | 0.0 |  | 60.0 |  | 0.0 |  | 54.5 |  |
|  | Bridge | 0.0 |  | 0.0 |  | 0.0 |  | 27.3 |  |
| telophase I | Normal | 100.0 | 1 | n.a. | 0 | 100.0 | 1 | 100.0 | 6 |
| Interkinesis | Normal | 100.0 | 12 | 100.0 | 4 | 100.0 | 5 | 91.7 | 12 |
|  | Bridge | 0.0 |  | 0.0 |  | 0.0 |  | 8.3 |  |
| metaphase II | Normal | 100.0 | 71 | 100.0 | 1 | 100.0 | 2 | 75.0 | 4 |
|  | Lack of orientation | 0.0 |  | 0.0 |  | 0.0 |  | 25.0 |  |
| anaphase II | Normal | 88.2 | 17 | n.a. | 0 | 100.0 | 2 | 100.0 | 3 |
|  | Laggards | 11.8 |  | n.a. |  | 0.0 |  | 0.0 |  |
| telophase II | Normal | 100.0 | 4 | n.a. | 0 | 100.0 | 2 | 100.0 | 2 |

A

B

**Figure S3:** Representative images of chromosome dynamics in ‘Eti Kehel’ (BB) with (A) a chromosome bridge during anaphase I, and (B) a chromosome bridge during interkinesis. Scale bar; 10 µm.

**Table S6:** Quantification of meiotic defects in ‘Pisang Lilin’, ‘Pisang Jari Buaya’, ‘Enzirabahima’ and ‘Williams Bell’. For each variety, the aberration type as well as its relative abundance (%) is provided, together with the total amount of meiocytes observed in a certain meiotic stage (n).

| Meiotic stage | Defect type | AAcv | | | | AAA | | | |
| --- | --- | --- | --- | --- | --- | --- | --- | --- | --- |
|  |  | Pisang Lilin | | Pisang Jari Buaya | | Enzirabahima | | Williams Bell | |
|  |  | % | n | % | n | % | n | % | n |
| pachytene | Normal | 78.4 | 37 | 100.0 | 30 | 100.0 | 11 | 100.0 | 24 |
|  | Asynapsis | 21.6 |  | 0.0 |  | 0.0 |  | 0.0 |  |
| diakinesis | Normal | 43.5 | 23 | 80.8 | 26 | 0.0 | 3 | 33.3 | 18 |
|  | Univalents | 0.0 |  | 7.7 |  | 33.3 |  | 66.7 |  |
|  | Trivalents | 0.0 |  | 0.0 |  | 100.0 |  | 0.0 |  |
|  | Clusters | 56.5 |  | 11.5 |  | 66.7 |  | 0.0 |  |
| metaphase I | Normal | 91.0 | 78 | 65.7 | 32 | 0.0 | 6 | 0.0 | 2 |
|  | Univalents | 9.0 |  | 28.1 |  | 0.0 |  | 100.0 |  |
|  | Nonhomologous recombination | 0.0 |  | 3.1 |  | 100.0 |  | 0.0 |  |
|  | Lack of orientation | 0.0 |  | 3.1 |  | 0.0 |  | 0.0 |  |
| anaphase I | Normal | 70.0 | 20 | 21.4 | 14 | 0.0 | 6 | 0.0 | 2 |
|  | Univalents | 0.0 |  | 14.3 |  | 0.0 |  | 100.0 |  |
|  | Laggards | 25.0 |  | 28.6 |  | 50.0 |  | 0.0 |  |
|  | Bridge | 5.0 |  | 35.7 |  | 50.0 |  | 0.0 |  |
| telophase I | Normal | 100.0 | 3 | 100.0 | 3 | 100.0 | 2 | 100.0 | 2 |
| Interkinesis | Normal | 80.0 | 10 | 100.0 | 7 | 85.7 | 7 | 100.0 | 13 |
|  | Unequal division | 0.0 |  | 0.0 |  | 14.3 |  | 0.0 |  |
|  | No cytokinesis | 20.0 |  | 0.0 |  | 0.0 |  | 0.0 |  |
| metaphase II | Normal | 87.5 | 38 | 68.7 | 16 | 50.0 | 4 | 50.0 | 4 |
|  | Laggards | 7.9 |  | 0.0 |  | 0.0 |  | 0.0 |  |
|  | Early chromatid separation | 0.0 |  | 18.8 |  | 0.0 |  | 50.0 |  |
|  | Lack of orientation | 0.0 |  | 12.5 |  | 50.0 |  | 0.0 |  |
| anaphase II | Normal | 38.4 | 13 | 68.7 | 16 | 33.3 | 3 | 66.7 | 3 |
|  | Laggards | 23.1 |  | 31.3 |  | 66.7 |  | 33.3 |  |
|  | Bridge | 38.5 |  | 0.0 |  | 0.0 |  | 0.0 |  |
| telophase II | Normal | 50.0 | 2 | 100.0 | 12 | 100.0 | 3 | 0.0 | 3 |
|  | Laggards | 50.0 |  | 0.0 |  | 0.0 |  | 0.0 |  |
|  | Polyads | 0.0 |  | 0.0 |  | 0.0 |  | 100.0 |  |

B

A

**Figure S4:** Representative images of metaphase I chromosomes in ‘Pisang Jari Buaya’ (AAcv) with (A) nonhomologous recombination, and (B) Aberrant metaphase plate orientation. Scale bar; 10 µm.

a

a

a

a

C

D

b

a

B

a

a

A

**Figure S5:** Distributions of (A) mean pollen diameters (µm), (B) the amount of measured pollen, (C) the percentage of large pollen and (D) the percentage of small pollen for the AA and AAcv genome groups sampled in Tanzania. Significant differences according to Kruskal-Wallis tests are indicated by distinct letters in the graphs.

**Figure S6:** Pollen diameter (µm) distributions for genotypes obtained from Tanzania.

**Figure S7:** Pollen diameter (µm) distributions for genotypes obtained from Brazil.

**Figure S8:** Pollen diameter (µm) distributions for genotypes common between Tanzania ‘Tz’ and Brazil ‘Br’.

**Figure S9:** Pearson correlation test between the combned nuclear size (µm²) and pollen diameter (µm) measurements of two AA genotypes, one BB genotype, three AAcv genotypes, one AAA genotypes, one ABB genotype, one AAAA genotype and one AAAB genotype.

**Table S7:** Quantification of the percentage of pollen with nuclei in a certain size range (µm²) for different genotypes belonging to different genome groups.

| **Genome group** | **Genotype** | **Total nuclei** | **Aneuploid** | **Haploid** | **Diploid** | **Triploid** | **Tetraploid** |
| --- | --- | --- | --- | --- | --- | --- | --- |
|  |  |  | < 100 µm²  (%) | 100 - 300 µm²  (%) | 301 - 477 µm²  (%) | 478 - 615 µm²  (%) | 616 - 759 µm²  (%) |
| **AA** | Borneo | 61 | 0.0 | 100.0 | 0.0 | 0.0 | 0.0 |
|  | truncata | 14 | 7.1 | 85.7 | 7.1 | 0.0 | 0.0 |
| **BB** | Eti Kehel | 50 | 14.0 | 80.0 | 6.0 | 0.0 | 0.0 |
| **AAcv** | Malaysian Blood | 61 | 11.5 | 88.5 | 0.0 | 0.0 | 0.0 |
|  | Pisang Jari Buaya | 58 | 12.1 | 77.6 | 10.3 | 0.0 | 0.0 |
|  | Pisang Lilin | 72 | 1.4 | 58.3 | 33.3 | 4.2 | 2.8 |
| **AAA** | Yangambi Km5 | 36 | 0.0 | 75.0 | 19.4 | 2.8 | 2.8 |
| **ABB** | Benedetta | 39 | 0.0 | 89.7 | 10.3 | 0.0 | 0.0 |
| **AAAA** | Ambrosia | 28 | 3.6 | 39.3 | 50.0 | 7.1 | 0.0 |
| **AAAB** | Ouro Da Mata | 37 | 5.4 | 86.5 | 8.1 | 0.0 | 0.0 |

B

A

**Figure S10:** Pearson correlation tests between (A) the percentage of higher ploidy nuclei (nuclear area > 300 µm²) and the percentage of large pollen (diameter > 120 µm), and (B) the percentage of aneuploid nuclei (nuclear area < 100 µm²) and the percentage of small pollen (diameter < 60 µm).

**Table S8:** Characteristics of pollen grain distributions of the 71 genotypes sampled in Arusha, Tanzania, in September 2021, including their mean pollen diameter, standard deviation of the pollen diameter, the amount of measured pollen, the percentage of larger pollen (diameter > 120µm), and the amount of larger pollen found in literature. Data obtained from Ortiz (1997) is indicated with ^1^, from Sathiamoorthy (1994) with ^2^ and from Ssebuliba et al. (2008) with ³. When the percentage of larger pollen found was not specified, presence of larger pollen is indicated by >0.0%. A substantial deviation in the percentage of larger pollen from the percentage of larger pollen found in literature for an genotype, is marked with *.

| Species  /Group | Name | Mean pollen diameter (µm) | Measured  pollen  amount | Small pollen (%) | Large pollen (%) | Large pollen literature (%) |
| --- | --- | --- | --- | --- | --- | --- |
| AA | A3617-9 | 82.6±7.0 | 836 | 0.0 | 0.0 |  |
| AA | Agutay | 86.5±1.5 | 2 | 0.0 | 0.0 |  |
| AA | Banksii | 86.2±11.7 | 48 | 2.0 | 0.0 | 0.0^1^ |
| AA | Borneo | 85.3±5.3 | 322 | 0.0 | 0.0 | 0.0^1^ |
| AA | Calcutta4 | 83.4±7.1 | 3441 | 0.0 | 0.0 | 0.0^1^ |
| AA | Hybrid | 88.2±8.0 | 1018 | 0.0 | 0.0 | 0.0^1^ |
| AA | Khae(Phrae) | 96.9±13.3 | 153 | 0.0 | 5.2 |  |
| AA | LongTavoy | 102.9±10.5 | 1862 | 0.0 | 3.7 |  |
| AA | M.A. spp. banksii | 88.8±9.2 | 1378 | 0.0 | 0.7 | 0.0^1^ |
| AA | Madang | 93.5±6.1 | 3 | 0.0 | 0.0 |  |
| AA | MaiaOa | 102.1±15.8 | 290 | 0.3 | 14.8 |  |
| AA | Pa Musore no2 | 93.2±13.8 | 1659 | 0.0 | 5.4* | 0.0^1^ |
| AA | Pa Musore no3 | 85.9±5.6 | 321 | 0.0 | 0.0 | 0.0^1^ |
| AA | Pa Songkhla | 86.7±6.2 | 3222 | 0.0 | 0.0 | 0.0^1^ |
| AA | Pahang | 92.7±10.7 | 261 | 0.0 | 0.8 | 0.0^1^ |
| AA | Pisang CiciAlas | 82.8±8.0 | 2043 | 0.0 | 0.0 |  |
| AA | Pisang Kra | 88.6±7.1 | 951 | 0.1 | 0.0 |  |
| AA | Pisang Serun 400 | 87.1±6.8 | 2426 | 0.0 | 0.0 |  |
| AA | Pisang Serun 404 | 94.3±10.7 | 245 | 0.0 | 0.9 |  |
| AA | Selangor | 83.4±5.9 | 1523 | 0.0 | 0.0 | 0.0^1^ |
| AA | Selangor 2 | 85.2±6.1 | 1329 | 0.0 | 0.1 | 0.0^1^ |
| AA | Truncata | 96.7±14.3 | 184 | 0.0 | 5.97* | 0.0^1^ |
| AA | Zebrina G.F. | 89.4±9.6 | 220 | 0.0 | 0.0 | 0.0^1^ |
| AAcv | AA cvRose | 85.1±8.7 | 1326 | 0.0 | 0.01 |  |
| AAcv | Akondro Mainty | 99.1±11.0 | 158 | 0.0 | 3.8 |  |
| AAcv | Awondaeke | 80.1±9.4 | 1180 | 0.0 | 0.3 |  |
| AAcv | Binaktong | 91.1±10.1 | 247 | 0.0 | 0.8 |  |
| AAcv | EMBRAPA205 | 94.6±12.2 | 377 | 0.0 | 1.6 |  |
| AAcv | Garunga | 97.0±9.9 | 94 | 0.0 | 3.2 |  |
| AAcv | Guyod | 100.9±17.6 | 121 | 1.6 | 13.2 | >0.0^1^ |
| AAcv | Hova | 87.5±5.4 | 853 | 0.0 | 0.0 |  |
| AAcv | IjihuIkundu | 92.8±9.7 | 394 | 0.3 | 0.3 |  |
| AAcv | Kahuti | 94.0±11.9 | 183 | 0.0 | 1.6 |  |
| AAcv | Katual Vunalir | 86.5±6.6 | 166 | 0.0 | 0.0 |  |
| AAcv | Kluai Lep Mu Nang | 92.3±10.7 | 219 | 0.0 | 0.0 |  |
| AAcv | Kuspaka | 92.4±7.3 | 87 | 0.0 | 0.0 |  |
| AAcv | Kwaro | 103.5±17.7 | 93 | 1.1 | 20.4 |  |
| AAcv | Makyughu 1 | 87.6±8.1 | 30 | 0.0 | 0.0 |  |
| AAcv | Makyughu 2 | 95.9±15.7 | 128 | 0.0 | 5.5 |  |
| AAcv | Mala | 90.6±12.4 | 96 | 1.0 | 3.1 |  |
| AAcv | Maleb | 83.0±8.2 | 94 | 0.0 | 0.0 |  |
| AAcv | Malaysian Blood | 79.9±9.2 | 547 | 0.0 | 0.2 |  |
| AAcv | Mambee Thu | 85.6±7.1 | 242 | 0.0 | 0.4 |  |
| AAcv | Mshale | 94.4±11.2 | 318 | 0.3 | 1.6 |  |
| AAcv | Mshale Mlelembo | 96.5±12.4 | 68 | 0.0 | 4.4 |  |
| AAcv | Ngu | 116.8±17.5 | 172 | 0.6 | 41.3 |  |
| AAcv | Nzumohel | 96.7±15.1 | 186 | 0.5 | 5.9 |  |
| AAcv | Paka | 81.5±11.3 | 202 | 0.0 | 0.5 | 3.3^2^ |
| AAcv | Pamoti On | 81.8±12.9 | 3 | 0.0 | 0.0 |  |
| AAcv | Pisang Berlin | 91.1±9.1 | 954 | 0.3 | 0.0 |  |
| AAcv | Pisang Buntal | 98.2±8.5 | 29 | 0.0 | 0.0 |  |
| AAcv | Pisang Jari Buaya | 87.2±13.0 | 22 | 0.0 | 0.0* | >0.0^1^ |
| AAcv | Pisang Kermian | 84.4±6.9 | 672 | 0.0 | 0.0 |  |
| AAcv | Pisang Lilin | 102.2±15.4 | 267 | 0.0 | 11.6 | 8.5^1,3^ |
| AAcv | Pisang Madu | 84.7±9.5 | 246 | 0.0 | 0.4 | 0.0^1^ |
| AAcv | Pisang Pipit | 96.4±11.1 | 430 | 0.0 | 2.6 |  |
| AAcv | Pisang Sapon | 89.7±7.2 | 1064 | 0.0 | 0.0 |  |
| AAcv | Pitu | 84.8±9.5 | 555 | 0.0 | 0.4 | >0.0^1^ |
| AAcv | PuTeWey | 84.4±10.4 | 496 | 0.0 | 0.0 |  |
| AAcv | Racadang | 78.9±14.5 | 15 | 12.5 | 0.0 |  |
| AAcv | Sena | 86.3±7.1 | 1527 | 0.0 | 0.1 |  |
| AAcv | Sihir | 90.1±8.8 | 376 | 0.0 | 0.8 |  |
| AAcv | Sowmuk | 81.8±12.7 | 157 | 3.8 | 0.6 |  |
| AAcv | Tainga | 86.2±8.8 | 858 | 0.2 | 0.0 |  |
| AAcv | Too | 84.1±8.9 | 579 | 0.7 | 0.0 |  |
| AAcv | Tuldo Tumbaga | 88.7±14.2 | 6 | 0.0 | 0.0 |  |
| AAcv | Type2 | 100.1±9.6 | 404 | 0.0 | 2.5 |  |
| AAcv | Uwati | 96.2±14.13 | 297 | 0.3 | 5.7* | 0.0^1^ |
| AAcv | Waimara | 87.0±9.0 | 13 | 0.0 | 0.0 |  |
| AAcv | Yenai | 91.7±6.9 | 637 | 0.0 | 0.0 |  |
| BB | Eti Kehel | 91.1±15.3 | 611 | 1.0 | 4.1 |  |

**Table S9:** Characteristics of pollen grain distributions of the 31 genotypes sampled in Bahia, Brazil, in March 2022, including their mean pollen diameter, standard deviation of the pollen diameter, the amount of measured pollen, the percentage of larger pollen (diameter > 120µm), and the amount of larger pollen found in literature. Data obtained from Ortiz (1997) is indicated with ^1^ and from Sathiamoorthy (1994) with ^2^. When the percentage of larger pollen found was not specified, presence of larger pollen is indicated by >0.0%. A substantial deviation in the percentage of larger pollen from the percentage of larger pollen found in literature for an genotype, is marked with *.

| Species  /Group | Name | Mean pollen diameter (µm) | Measured  pollen  amount | Small pollen (%) | Large pollen (%) | Large pollen literature (%) |
| --- | --- | --- | --- | --- | --- | --- |
| AA | Calcutta 4 | 64.2±9.2 | 10715 | 33.0 | 0.0 |  |
| AA | Krasan Saichon | 85.7±9.4 | 3022 | 1.6 | 0.2 |  |
| AA | Malaccensis | 76.3±10.4 | 3638 | 0.1 | 0.8 |  |
| AA | Malbut | 79.1±7.5 | 94 | 0.0 | 0.0 |  |
| AA | Pa Musore 2 | 74.2±8.9 | 1858 | 0.1 | 0.6 |  |
| AA | Pa Rayong | 79.1±9.4 | 3576 | 2.0 | 0.0 |  |
| AA | Pisang Serun | 71.5±11.4 | 5655 | 15.2 | 0.1 |  |
| AA | Selangor | 76.1±12.0 | 1373 | 6.8 | 0.3 | 0.0^1^ |
| AAcv | IDO110 | 75.7±11.2 | 936 | 6.9 | 0.0 | 0.0^1^ |
| AAcv | Pa Patthalung | 90.4±21.3 | 44 | 9.1 | 4.5 | 0.0^1^ |
| AAcv | Pisang Kermain | 84.8±25.9 | 415 | 21.0 | 7.9 |  |
| AAcv | Pisang Lidi | 82.9±11.8 | 1436 | 0.1 | 0.9 |  |
| AAcv | Pisang Lilin | 77.0±13.7 | 251 | 10.4 | 0.8* | 8.5^1,3^ |
| AAcv | Uwati | 92.0±12.8 | 70 | 4.3 | 0.0 |  |
| BB | BB Franca | 73.9±11.4 | 3220 | 9.5 | 0.2 | 0.0^2^ |
| BB | Balbisiana Franca | 74.3±10.1 | 3323 | 4.9 | 0.2 | 0.0^2^ |
| BB | Butuhan | 69.3±7.9 | 3686 | 12.0 | 0.0 | 0.0^1^ |
| AAA | Grande Naine | 108.0±25.0 | 6 | 0.0 | 16.7 |  |
| AAA | Khai Tong Ruang | 91.2±16.7 | 297 | 5.4 | 2.4 |  |
| AAA | Khai Tong Ruong | 91.8±16.3 | 178 | 4.5 | 3.4 |  |
| AAA | Yangambi Km5 | 93.6±18.2 | 529 | 5.9 | 6.6 | >0.0^1^ |
| AAB | Yangambi N2 | 123.7±40.0 | 6 | 0 | 33.3 |  |
| ABB | Benedetta | 86.2±17.6 | 180 | 0 | 3.9 |  |
| ABB | Blue Java | 85.5±15.5 | 96 | 3.1 | 3.1 |  |
| ABB | Moenang | 76.5±20.1 | 35 | 22.9 | 2.8 |  |
| ABB | Pisang Kepok Bung | 89.0±18.6 | 128 | 6.3 | 4.7 |  |
| ABB | Saba Honduras | 105.1±22.9 | 25 | 4.0 | 28.0* | 0.0^1^ |
| AAAB | BRS Platina | 109.0±18.2 | 228 | 0.4 | 22.4 |  |
| AAAB | BRS Princesa | 97.5±17.2 | 697 | 3.2 | 7.9 |  |
| AAAB | FHIA 18 | 83.2±25.2 | 36 | 25.0 | 0.0 |  |
| AAAB | Ouro Da Mata | 89.4±23.4 | 30 | 6.7 | 13.3* | 0.0^1^ |
